# Genomic analyses elucidate *S*‐locus evolution in response to intra‐specific losses of distyly in *Primula vulgaris*

**DOI:** 10.1002/ece3.10940

**Published:** 2024-03-21

**Authors:** E. Mora‐Carrera, R. L. Stubbs, G. Potente, N. Yousefi, B. Keller, J. M. de Vos, P. Szövényi, E. Conti

**Affiliations:** ^1^ Department of Systematic and Evolutionary Botany University of Zurich Zurich Switzerland; ^2^ Department of Environmental Sciences – Botany University of Basel Basel Switzerland

**Keywords:** hemizygosity, heterostyly, mating‐system transitions, *Primula*, *S*‐locus

## Abstract

Distyly, a floral dimorphism that promotes outcrossing, is controlled by a hemizygous genomic region known as the *S*‐locus. Disruptions of genes within the *S*‐locus are responsible for the loss of distyly and the emergence of homostyly, a floral monomorphism that favors selfing. Using whole‐genome resequencing data of distylous and homostylous individuals from populations of *Primula vulgaris* and leveraging high‐quality reference genomes of *Primula* we tested, for the first time, predictions about the evolutionary consequences of transitions to selfing on *S*‐genes. Our results reveal a previously undetected structural rearrangement in *CYPᵀ* associated with the shift to homostyly and confirm previously reported, homostyle‐specific, loss‐of‐function mutations in the exons of the *S*‐gene *CYPᵀ*. We also discovered that the promoter and intronic regions of *CYPᵀ* in distylous and homostylous individuals are conserved, suggesting that down‐regulation of *CYPᵀ* via mutations in its promoter and intronic regions is not a cause of the shift to homostyly. Furthermore, we found that hemizygosity is associated with reduced genetic diversity in *S*‐genes compared with their paralogs outside the *S*‐locus. Additionally, the shift to homostyly lowers genetic diversity in both the *S*‐genes and their paralogs, as expected in primarily selfing plants. Finally, we tested, for the first time, long‐standing theoretical models of changes in *S*‐locus genotypes during early stages of the transition to homostyly, supporting the assumption that two copies of the *S*‐locus might reduce homostyle fitness.

## Abstract 2

Here, we focus the transition from distyly to homostyly in the common primroses to study the causes and consequences of mating‐system transitions. Using whole‐genome resequencing data, we find that loss‐of‐functions mutations in the *CYP* ^*T*^ gene is associated with multiple independent transitions to homostyly and that this transitions has consequences in the so‐called distyly *S*‐locus. Finally, we tested, for the first time, long‐standing theoretical models of changes in *S*‐locus genotypes during early stages of the transition to homostyly.

## 1 INTRODUCTION

The repeated shift from outcrossing to selfing is a central topic in plant evolution (Cutter, 2019; Stebbins, 1957). The significance of this transition lies in its central role in altering the partitioning of genetic diversity within and among populations, thus influencing how populations respond to natural selection (Barrett, 2019; Wright et al., 2013). Additionally, mating‐system shifts can affect species longevity, with outcrossing species being more resilient and selfing species more prone to extinction (de Vos et al., 2014; Goldberg et al., 2010). Previous studies used phenotypic traits typically associated with selfing to estimate, for example, the number and tempo of transitions to selfing in phylogenies of ancestrally outcrossing taxa (de Vos et al., 2014; Goldberg & Igic, 2012). However, missing knowledge of the genes that control mating systems has hindered the study of molecular processes associated with transitions to selfing until recently, especially in non‐model organisms. Current advances in genomics now facilitate the identification of the genes and mutations associated with mating‐system shifts.

**FIGURE 1:**
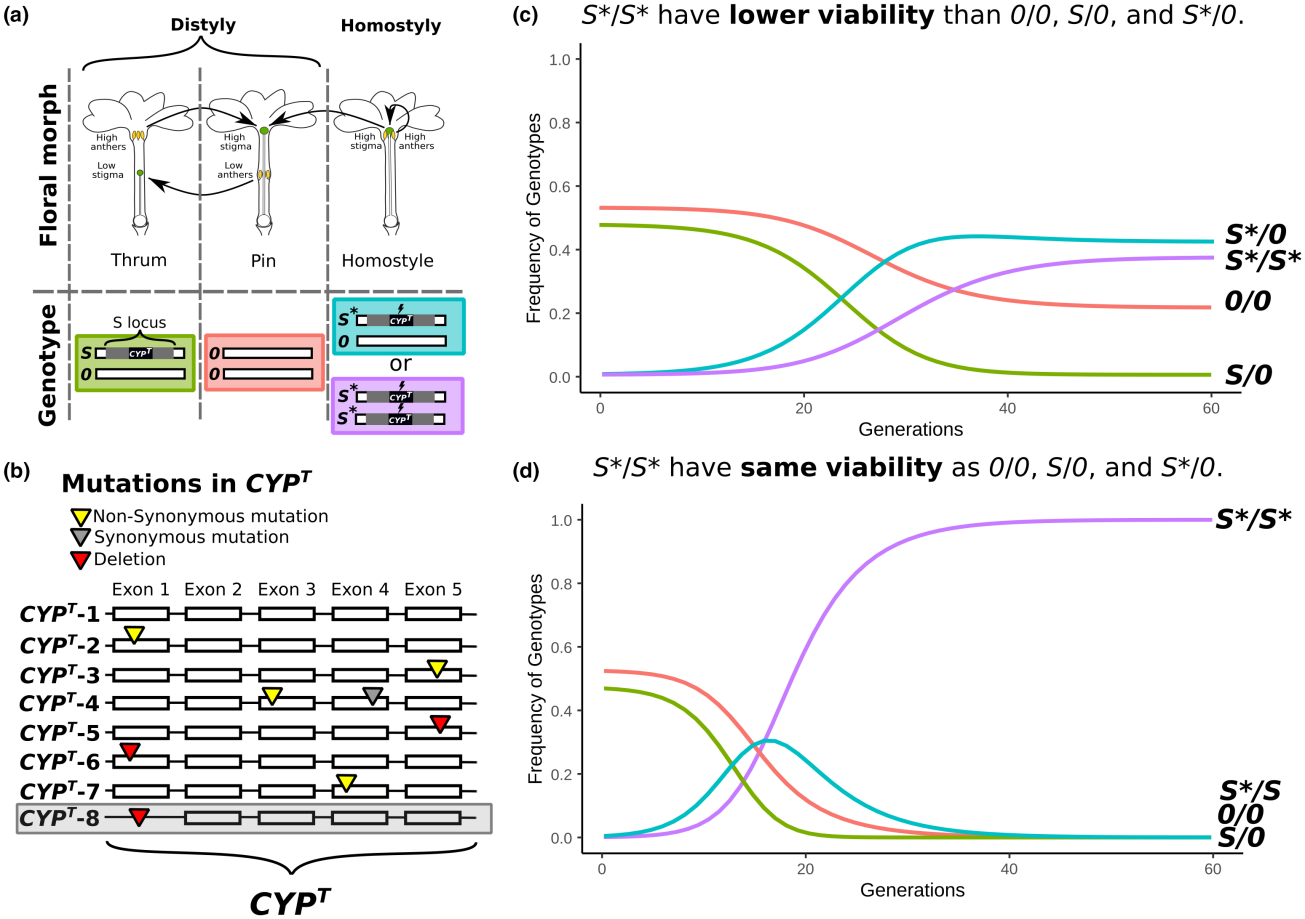
Distyly and homostyly in *Primula vulgaris*. (a) Phenotypes (top) and genotypes (bottom) of distylous (thrum; pin) and homostylous individuals in *P. vulgaris*. Short‐styled flowers (thrums) have male sexual organs (anthers) above the short female sexual organ (style), while long‐styled flowers (pins) have anthers below the long style. The reciprocity of sexual organs promotes pollen transfer between floral morphs, while self‐ and intra‐morph self‐incompatibility prevents selfing. Distyly is controlled by the *S*‐locus, which is hemizygous in thrums [*S*/0] and absent in pins [0/0]. Homostyly arises primarily through the disruption of the *CYP* ^*T*^ gene located in the *S*‐locus, causing both stigma elongation and loss of self‐incompatibility. Homostyles can have one [S*/0] or two copies [S*/S*] of the *S*‐locus with a disrupted *CYP* ^*T*^; *S*‐locus haplotypes with disrupted *CYP* ^*T*^ are designated with S*. Black arrows indicate compatible pollen transfers: distyly promotes outcrossing, homostyly enables selfing. (b) Graphical representation of the seven *CYP* ^*T*^ alleles from natural populations of *P. vulgaris* in Somerset, England, reported in Mora‐Carrera et al. (2021): *CYP* ^*T*^‐1 designates the functional copy in thrums and *CYP* ^*T*^‐2 to 7 designate alleles with deletions (red triangles), non‐synonymous (yellow triangles), and synonymous (gray triangles) mutations in different exons of the homostyles. The *CYP* ^*T*^‐8 allele (gray box), first reported here, is characterized by the loss of exon 1. (c) Predictions of changing frequencies of *S*‐locus genotypes over generations assuming that homostyles with S*/S*‐genotypes have 35% lower viability than thrums, pins, and S*/0‐homostyles. Equilibrium is reached when homostyles with S*/S*‐ vs. S*/0‐genotypes segregate at roughly equal frequencies, while pin genotypes (0/0) are maintained at low frequencies and thrum genotypes (S/0) disappear from the population (c). (d) Predictions of changing frequencies of *S*‐locus genotypes over generations assuming that S*/S*‐homostyles have equal viability as thrums, pins, and S*/0‐homostyles (see Table S1). Equilibrium is reached when S*/S*‐homostyles become fixed in the population. Plots in (c) and (d) were generated in R v3.6 using original equations of Crosby's model (Crosby, 1949). At the phenotypic level, the model predicts a sharp increase of homostyles over generations at the expense of thrums, with pins remaining either at low frequencies (1c) or disappearing (1d). Calculating the actual counts of S*/S*‐ and S*/0‐genotypes in natural populations had been impossible until recently because discriminating between the two types of homostylous genotypes requires knowledge of *S*‐locus genes and ability to determine whether the *S*‐locus is haploid or diploid (see Table 4).

A prime model to investigate the transition from outcrossing to selfing has been the shift from distyly to homostyly in *Primula* (Barrett, 2019). Distyly is characterized by the co‐occurrence in populations of two floral types (called the pin and thrum, respectively) of self‐incompatible individuals distinguished by the reciprocal arrangement of male (anthers) and female (stigma) sexual organs in their flowers (Figure 1a; Ganders, 1979; Keller et al., 2014; Lloyd & Webb, 1992). Specifically, pins are characterized by having the stigma positioned above the anther level within the flowers, whereas thrums have the anthers above the stigma level. This floral heteromorphism represents an adaptation for outcrossing reported in at least 26 angiosperm families (Naiki, 2012). Conversely, homostyly is a floral homomorphism that enables selfing. It is characterized by self‐compatible individuals bearing flowers with both stigma and anthers at the same level in the corolla tube (Figure 1a; Barrett, 2019). Evidence supporting higher selfing in homostylous than distylous plants has been reported both intra‐ and inter‐specifically (Mora‐Carrera et al., 2021; Schoen et al., 1997; Zhao et al., 2023; Zhong et al., 2019). Additionally, independent shifts from distyly to homostyly have been documented both within and between species (Costa et al., 2019; de Vos et al., 2014; Kissling & Barrett, 2013; Ruiz‐Martín et al., 2018; Zhou et al., 2012).

It has long been known that the *S*‐locus, a supergene, controls distyly and the shift to homostyly (Lewis & Jones, 1992). However, the molecular and functional characterization of the *S*‐locus has been achieved only recently. The breakthrough occurred in *Primula*, where the *S*‐locus comprises five genes known as *S*‐genes (*CCM* ^*T*^, *GLO* ^*T*^, *CYP* ^*T*^, *PUM* ^*T*^, and *KFB* ^*T*^) and is hemizygous in thrums (*S*/0) but absent in pins (0/0; Figure 1a; Li et al., 2016; Potente, Léveillé‐Bourret, et al., 2022). Four of the five *S*‐genes (i.e., *CCM* ^*T*^, *GLO* ^*T*^, *CYP* ^*T*^, and *KFB* ^*T*^) are the product of gene duplications, and their paralogs (named *CCM1*, *GLO1*, *CYP734A51*, and *KFB1*) have been localized in *Primula veris*, where they are scattered throughout the genome (Potente, Léveillé‐Bourret, et al., 2022). The function of *S*‐genes has been experimentally characterized only for *GLO* ^*T*^ and *CYP* ^*T*^, which were recently shown to control key traits in thrum flowers: *GLO* ^*T*^ determines long anthers, while *CYP* ^*T*^ determines short stigma and female self‐incompatibility (Huu et al., 2016, 2020, 2022). Specifically, experimental silencing of *GLO* ^*T*^ in *Primula forbesii* thrums lowered anther position, producing flowers with both anthers and stigma in the middle of the corolla tube (i.e., short‐homostyly). However, self‐incompatibility was retained, preventing self‐fertilization in short‐homostyles (Huu et al., 2020). Conversely, silencing of *CYP* ^*T*^ in *P. veris* was associated with both style elongation and loss of self‐incompatibility, thus turning self‐incompatible thrum flowers into self‐compatible, homostylous flowers with both stigma and anthers at the mouth of the corolla tube (i.e., long‐homostyly; Huu et al., 2016, 2022). Although both short‐ and long‐homostyly have been reported, long‐homostyly is more common in *Primula* (Charlesworth & Charlesworth, 1979; Lewis & Jones, 1992). The likely explanation is that, as reported above, *CYP* ^*T*^ disruption causes both reduced herkogamy and self‐compatibility (Huu et al., 2016, 2020, 2022), thus enabling self‐pollination and the production of selfed seed in long‐homostylous flowers (Piper et al., 1986), whereas short‐homostyles with disrupted *GLO* ^*T*^, but functional *CYP* ^*T*^, cannot produce selfed seed despite their reduced herkogamy, for they retain self‐incompatibility (Huu et al., 2022). Given the higher frequency of long‐homostyles and the rarity of short homostyles in *Primula*, for simplicity's sake, we hereafter refer to long‐homostyly as homostyly (Figure 1a).

The shift from distyly to homostyly has been intensely studied in populations of *P. vulgaris* from Somerset, England, that display frequency variation of thrums, pins, and homostyles (Crosby, 1940, 1949). Targeted Sanger sequencing of the five *CYP* ^*T*^ exons revealed that all tested thrums from the mentioned region shared the same functional *CYP* ^*T*^ allele (*CYP* ^*T*^‐1; Figure 1b), whereas 21 homostyles harbored six different *CYP* ^*T*^ alleles, each with a unique, potentially disruptive mutation (*CYP* ^*T*^‐2 to *CYP* ^*T*^‐7 in Mora‐Carrera et al., 2021; Figure 1b). One possible explanation for the lack of shared *CYP* ^*T*^ mutations among the homostyles is that homostyly evolved independently multiple times. However, the same study also found that six homostyles from two different populations had the same *CYP* ^*T*^ allele as that of thrums (i.e., *CYP* ^*T*^‐1). This result raised the possibility that homostyly initially arose via *CYP* ^*T*^ silencing caused by either a structural rearrangement (e.g., inversion or translocation) involving any of the *CYP* ^*T*^ exons or an inactivating mutation in the *CYP* ^*T*^ promoter or a mutation in one of the intronic regions disrupting exon processing, subsequently followed by multiple, unique mutations in *CYP* ^*T*^ exons, as those found in *CYP* ^*T*^‐*2* to *CYP* ^*T*^‐*7* (Charlesworth, 2022; Mora‐Carrera et al., 2021). The three types of mutations mentioned above cannot be detected using data from Sanger sequencing of individual *CYP* ^*T*^ exons but can be detected by analyzing data from Whole Genome Resequencing (WGR). Furthermore, determining whether homostyly in *P. vulgaris* arose multiple times via independent mutations in *CYP* ^*T*^ exons or once through any of the three mentioned types of mutations requires a high‐quality annotation of the *S*‐genes in a reference genome from the same or a closely related species and WGR data covering coding and non‐coding regions of *CYP* ^*T*^. Both types of resources, that is, WGR and reference genomes, are now available, enabling an expanded analysis of the molecular mechanisms possibly causing the transition to homostyly in *P. vulgaris*.

The recently published high‐quality annotation of the five *S*‐genes and their four paralogs (Potente, Léveillé‐Bourret, et al., 2022), combined with newly generated sequences of these nine genes extracted from WGR data of thrums and homostyles, provide an ideal opportunity to test how the genetic architecture of the *S*‐locus and the transition to homostyly affect genetic variation and selection on *S*‐genes and their paralogs. First, the thrum‐specific segregation of the hemizygous *S*‐locus should cause a 3/4th reduction of effective population size (Ne), lowering genetic diversity in *S*‐genes (Huu et al., 2016). Moreover, hemizygosity could have contrasting effects on the efficacy of purifying selection on *S*‐genes. On the one hand, the reduction of *N_e_* in the *S*‐genes should make purifying selection less efficient when compared to their paralogs (Huu et al., 2016). On the other hand, similarly to what happens in the Y sex‐chromosome (Gossmann et al., 2011), selection to maintain function of *S*‐genes, and the exposure of recessive deleterious mutations under hemizygosity should enhance the efficacy of purifying selection when compared to their paralogs. Second, the transition to homostyly should reduce genetic diversity in both *S*‐genes and their paralogs due to increased homozygosity in homostyles (Mora‐Carrera et al., 2021). However, the extent to which the efficacy of purifying selection differs between *S*‐genes and their paralogs and whether the transition to homostyly reduces genetic diversity in these genes remain poorly understood (Potente, Léveillé‐Bourret, et al., 2022).

Additionally, the WGR data newly presented here allowed us to assess the ploidy level of specific genomic regions via the analysis of read depth (Gutiérrez‐Valencia et al., 2022), thus enabling the testing of long‐standing predictions, first proposed by Crosby (1949), regarding the changing frequencies of *S*‐locus genotypes during the transition from distyly to homostyly. In a pioneering study, Crosby (1949) proposed a model for how the frequencies of thrums, pins, and homostyles change over time (Figure 1c,d). The model developed by Crosby (1949) assumed that thrums were typically heterozygous dominant at the *S*‐locus, pins were homozygous recessive, and homostyles stemmed from thrums via recombination at the *S*‐locus (Bateson & Gregory, 1905). Crosby assumed that the viability (i.e., the proportion of germinated seeds that developed into a seed‐producing plant) of homozygous homostyles was either lower than or equal to the viability of pins, thrums, and heterozygous homostyles. Crosby's assumption of lower viability for homozygous homostyles rested on previous crossing experiments by Mather and Winton (1941) suggesting that homozygous dominant thrums (*S*/*S*) had lower viability than heterozygous thrums. His model is applicable also under the recently demonstrated hemizygosity of the *S*‐locus in *Primula* by assuming that the viability of homostyles with a diploid disrupted *S*‐locus (S*/S*‐genotypes) is either 35% lower than or equal to the viability of homostyles with a haploid *S*‐locus (S*/0‐genotype), thrums (*S*/0‐genotype), and pins (0/0‐genotype; see Figures 1c,d).

In *P. vulgaris*, repeated phenotypic surveys conducted in Somerset, England, have shown that, when homostyles are present at high frequency, thrums tend to be less frequent and, in some cases, absent, compared with pins (Crosby, 1949; Curtis & Curtis, 1985; Mora‐Carrera et al., 2021). These findings align with the predictions of Crosby's model under lower viability of S*/S*‐homostyles (Figure 1c). However, one Somerset population consisted exclusively of homostyles (Curtis & Curtis, 1985; Mora‐Carrera et al., 2021), suggesting that the fixation of homostyly is possible, as expected under the model with equal viability for S*/S*‐ and S*/0‐homostyles. Of note, previous phenotypic surveys could not discriminate between S*/S*‐ and S*/0‐genotypes for homostyles due to the lack of adequate sequencing tools and molecular knowledge of the *S*‐locus. Recently developed sequencing technologies enable the estimation of sequencing depth at the *S*‐locus (Gutiérrez‐Valencia et al., 2022), allowing us to determine whether the *S*‐locus is haploid or diploid in homostylous and thrum individuals (the *S*‐locus being absent from pins). Therefore, it is now possible to estimate whether the observed frequencies of S*/S*‐ and S*/0‐homostyles in natural populations support the model assuming lower or equal viability for S*/S*‐genotypes in relation to the other genotypes, testing Crosby's genotypic predictions for the first time.

Here, we analyze new WGR data from nine populations of *P. vulgaris* with varying frequencies of pins, thrums, and homostyles, to answer the following questions: (1) Do all homostyles carrying different disrupted *CYP* ^*T*^ exons share the same mutation in its promoter region, or in one of the *CYP* ^*T*^ introns, and/or a structural rearrangement involving *CYP* ^*T*^ exons that might disrupt *CYP* ^*T*^ function or expression, allowing for the possibility of a single origin of homostyly? (2) Do the haploid *S*‐genes have lower genetic diversity and efficacy of purifying selection than their diploid paralogs, which are located outside the *S*‐locus? (3) Do *S*‐genes and their paralogs have lower genetic diversity in homostyles than in thrums? (4) Do observed frequencies of S*/0‐ and S*/S*‐homostyles in natural populations better match genotypic frequencies predicted under the assumptions of lower or equal viability for S*/S*‐homostyles? Our study illustrates how recently acquired knowledge of the genes controlling mating systems combined with high‐quality genomic resources can generate novel insights into the genotypic changes and evolutionary consequences associated with phenotypic transitions from outcrossing to selfing.

## 2 MATERIALS AND METHODS

### 2.1 Study species

*Primula vulgaris* (primrose) is an ancestrally heterostylous (de Vos et al., 2014; Mast et al., 2006), diploid (2*n* = 22), perennial, rosette‐forming plant blooming from February to April. Distylous populations of *P. vulgaris* occur across Eurasia, including in Turkey, most of Western Europe, larger Mediterranean Islands, and all British Isles (Jacquemyn et al., 2009). In contrast, populations with varying frequencies of homostyles have been discovered only in Somerset and Chiltern Hills, England (Crosby, 1940, 1949), and one population in the Netherlands (Barmentlo et al., 2017). Habitat fragmentation due to intensive pastoral and agricultural activities in these areas were suggested as the potential selective pressure favoring the shift to self‐fertilizing homostyles in these populations (Mora‐Carrera et al., 2021).

### 2.2 Sampling and sequencing of plant material

In spring 2019, we collected leaf tissue from 105 individuals of *P. vulgaris*, comprising 74 heterostyles (37 pins and 37 thrums) and 31 homostyles. The samples were obtained from nine populations, including: six dimorphic populations (comprising only pins and thrums) from Turkey (TR‐D), Slovakia (SK‐D), Switzerland (CH‐D) and England (EN1‐D, EN2‐D, and EN3‐D); two trimorphic populations (comprising pins, thrums, and homostyles) from England (EN4‐T and EN5‐T); and one monomorphic population (comprising only homostyles) from England (EN6‐M) (Table 1). Populations EN4‐T, EN5‐T, and EN6‐M corresponded to populations T04, T07, and M01, respectively, from our previous study (Mora‐Carrera et al., 2021). Moreover, the 11 homostyles from EN6‐M analyzed here included three of the homostyles carrying *CYP* ^*T*^‐1 (detected with Sanger sequencing) from population M01 of Mora‐Carrera et al. (2021; Figure 1b).

**TABLE 1:** Summary of sampled *Primula vulgaris* populations, collected individuals, and frequencies of *S*‐locus genotypes (*S*/0 or *S*/*S*, 0/0, S*/0, and S*/S*).

Population	Latitude	Longitude	Population type	Collected individuals (thrum:pin:homostyle)	Thrum	Pin	Homostyle	
					*S*/0: *S*/*S*	0/0	S*/0	S*/S*
TR‐D	40.74	39.55	D	5:5:–	0.5	0.5	–	–
SK‐D	48.77	19.05	D	5:5:–	0.5	0.5	–	–
CH‐D	46.44	6.91	D	5:5:–	0.5	0.5	–	–
EN1‐D	52.14	−0.13	D	5:4:–	0.55	0.45	–	–
EN2‐D	51.35	−2.33	D	4:5:–	0.22:0.22	0.55	–	–
EN3‐D	51.09	−2.71	D	5:5:–	0.5	0.5	–	–
EN4‐T	51.08	−2.30	T	3:4:10	0.17	0.24	0.24	0.35
EN5‐T	51.13	−2.42	T	4:5:10	0.16:0.04	0.27	0.32	0.21
EN6‐M	51.03	−2.63	M	–:–:11	–	–	–	1

*Note*: Ploidy level (haploid vs. diploid) of *S*‐locus genotypes was identified by estimating the sequencing depth of *S*‐locus genes relative to the sequencing depth of genome‐wide coding regions. Population abbreviations—geographic origin: CH, Switzerland; EN, England; SK, Slovakia; TR, Turkey; digit following geographic acronym indicates population number; capital letter following dash indicates: D, dimorphic population with pins and thrums; M, monomorphic population consisting entirely of homostyles; T, trimorphic population with pins, thrums, and homostyles.

To investigate possible molecular mechanisms for the transition to homostyly not detected before and the consequences of such transition on *S*‐genes and their paralogs in *P. vulgaris*, we generated WGR data for all the above 105 individuals, as follows. DNA extractions were performed using the Maxwell extraction method (Promega, USA) at the Functional Genomics Center Zurich (Zurich, Switzerland). Library preparation and paired‐end sequencing (150 bp) were conducted by RAPiD GENOMICS (Gainesville, Florida, USA) using Illumina Novaseq6000 platform, generating 7,077,866,510 paired‐end sequencing reads with an average sequencing depth of 18.9 (±SD = 3.08).

### 2.3 Mapping and variant calling of WGR data to *CYP* ^*T*^ of the *P. vulgaris* genome

To determine the sequences of all five exons and four introns of *CYP* ^*T*^, along with its up‐ and down‐stream intergenic regions, we first produced a high‐quality reference *S*‐locus assembly for *P. vulgaris* by replacing all *S*‐locus contigs (LH_v2_0002458, LH_v2_0067593, LH_v2_0003915, and LH_v2_0000241) in the genome assembly of *P. vulgaris* (Cocker et al., 2018) with a published, highly contiguous 450 kb sequence of the *P. vulgaris S*‐locus (Li et al., 2016). We then mapped WGR reads from all 105 individuals of *P. vulgaris* to the *S*‐locus assembly of the same species generated above. To determine the position of *CYP* ^*T*^ in the reference *S*‐locus assembly, we aligned the coding sequence of *CYP* ^*T*^ against the reference using the *query* function of blastn with default parameters, which is part of the NCBI BLAST+ toolkit v2.6.0 (Camacho et al., 2009). The described approach enabled accurate infra‐specific alignment of WGR reads to *CYP* ^*T*^ intronic and intergenic regions, which are expected to be highly variable because they are under lower selection than exonic regions. Prior to mapping, Illumina adapters were clipped from raw reads with Trimmomatic v0.38 (Bolger et al., 2014) using default parameters. Mapping was performed using BWA‐mem v7.17 (Li & Durbin, 2009) with default parameters. As negative control, pin individuals (0/0) were included in the analysis and, as expected, none of the sequencing reads from the 37 pins mapped to the *S*‐locus. Duplicated reads were marked with the MarkDuplicates tool included in Picard v2.18.4 (http://broadinstitute.github.io/picard/). Variant calling of SNPs and of insertions and deletions (indels) for the *S*‐locus was conducted using HaplotypeCaller, implemented in the Genome Analysis Toolkit (GATK) v4.1.2.0 (McKenna et al., 2010) pipeline. Finally, SNP variants were filtered from the Variant Call Format (VCF) file using the SelectVariants with the following filters: quality‐by‐depth (QD) > 2.0; mapping quality (MQ) > 40.0; strand bias (FS) < 60.0; mapping quality rank‐sum test (MQRankSum) > −12.5; a rank‐sum test (ReadPositionRankSum) > −8.0; site read depth (DP > ½X) || (DP < 3X). Additionally, sites with fixed heterozygosity (i.e., InbreedingCoeff < −0.99), likely representing incorrect SNP calling (O'Leary et al., 2018; Pavan et al., 2020), were filtered out.

### 2.4 Identification of mutations putatively disrupting *CYP* ^*T*^ function in *P. vulgaris*

#### 2.4.1 Disruptive mutations in *CYP* ^*T*^ coding regions

To identify potential homostyle‐specific, loss‐of‐function *CYP* ^*T*^ mutations, including non‐synonymous mutations, insertions, and deletions, we compared the sequences of the five *CYP* ^*T*^ exons in 31 homostyles with the functional *CYP* ^*T*^ allele of the 37 thrums. For this, we extracted the respective sequences of the five *CYP* ^*T*^ exons from the *S*‐locus VCF file using the *intersect* function included in BEDtools v2.29.2 (Quinlan & Hall, 2010) and converted them into a single FASTA file using vcf2phylip.py (https://github.com/edgardomortiz/vcf2phylip). The sequence alignment of all exons and the detection of putatively disruptive mutations in *CYP* ^*T*^ of homostyles and thrums were performed with MEGA X (Kumar et al., 2018). Finally, we compared the resulting sequence alignment with an alignment of previously detected mutations in *CYP* ^*T*^ exons reported by Mora‐Carrera et al. (2021).

#### 2.4.2 Mutations in *CYP* ^*T*^ intronic regions

To determine whether the shift to homostyly is associated with mutations involving *CYP* ^*T*^ introns that might affect intron splicing (causing, e.g., inactivating reading frameshifts), we examined the alignment of all four introns in the sampled thrums (37 individuals) and homostyles (31 individuals) to detect any single‐nucleotide polymorphism (SNP) fixed in the homostyles but absent in thrums.

#### 2.4.3 Structural rearrangements in *CYP* ^*T*^

To determine whether the shift to homostyly is associated with structural rearrangements involving *CYP* ^*T*^ exons, we examined the paired‐end sequencing reads mapped to the introns and exons of *CYP* ^*T*^ in both thrums (as a reference) and homostyles using the Interactive Genomic Viewer (IGV) v2.8.6 (Robinson et al., 2011). Translocations can be identified by analyzing the mapped paired‐end reads, where one read is mapped to one position in the genome (e.g., *CYP* ^*T*^ in the *S*‐locus), while its mate‐pair is mapped to a different position, either in the same or different chromosome. Inversions can be detected by comparing the orientation of the mapped read‐pairs to *CYP* ^*T*^ in the reference genome. If there is a small inversion, both mapped paired‐end reads should be oriented in the same direction (→→ or ←←), whereas in the absence of a structural change normal mapped paired‐end reads should be oriented toward each other (→←). Finally, deletions are characterized by drops in sequencing read coverage at specific positions in the genome, in this case, within *CYP* ^*T*^. We characterized a deletion as a complete absence of mapping depth and a site read depth equal to zero (DP = 0).

#### 2.4.4 Disruptive mutations in *CYP* ^*T*^ promoter region

To determine whether mutations in the promoter region are responsible for *CYP* ^*T*^ loss of function in homostyles, we conducted an analysis to identify the putative *CYP* ^*T*^ transcription‐factor binding‐site and searched it for homostyle‐specific mutations. It is expected that the 3 kb region upstream of a gene of interest contains its promoter, including the transcription‐factor binding‐site (Yu et al., 2016); thus, we first extracted and aligned the 3 kb sequence upstream of *CYP* ^*T*^ exon 1 from 20 thrums in dimorphic populations TR‐D, SK‐D, CH‐D, and EN1‐D of *P. vulgaris*. Considering that the transcription‐factor binding‐site important for distyly should be relatively conserved across species, we included the 3 kb sequence upstream of *CYP* ^*T*^ from the reference genome of *P. veris* (Potente, Léveillé‐Bourret, et al., 2022) to our aligned sequence from the 20 individuals of *P. vulgaris*. To identify motifs enriched for transcription‐factor binding‐sites in the aligned dataset of relevant sequences from 20 thrums of *P. vulgaris* and one *P. veris* individual (see above), we employed the Simple Enrichment Analysis (SEA) implemented in the Multiple Em for Motif Elicitation (MEME) suite program v5.5.0 (McLeay & Bailey, 2010). We used the *Arabidopsis thaliana* DAP motifs database (O'Malley et al., 2016) and default parameters to identify the aforementioned enriched motifs. Finally, after identifying enriched motif sequences in thrums, we compared all 37 thrums and 31 homostyles by aligning the enriched motifs using MEGA X (Kumar et al., 2018) and inspected the alignment to check whether any SNP was fixed in the homostyles but absent in thrums. To determine the potential function of the identified enriched motifs, we consulted The *Arabidopsis* Information Resource (TAIR) (http://www.arabidopsis.org).

In addition to detecting transcription‐factor binding‐sites, we searched for additional *cis*‐regulatory elements in the 3 kb region upstream of *CYP* ^*T*^ using PlantCARE (Lescot et al., 2002) as performed in Henning et al. (2023). For this analysis, we obtained the sequences of the 3 kb upstream of *CYP* ^*T*^ from the reference *S*‐locus genomes of *P. vulgaris* (Cocker et al., 2018) and *P. veris* Potente, Léveillé‐Bourret, et al., 2022). Once the putative *cis*‐regulatory elements were identified, we searched for mutations in these elements that were fixed in homostyles but absent in thrums.

### 2.5 Genetic variation in *S*‐genes and their paralogs

To quantify genetic variation in the five *S*‐genes (*CCM* ^*T*^, *GLO* ^*T*^, *CYP* ^*T*^, *PUM* ^*T*^, and *KFB* ^*T*^) and their four paralogs (*CCM1*, *GLO1*, *CYP734A51*, and *KFB1*), we calculated nucleotide diversity (π) at synonymous (π_S_) and non‐synonymous (π_N_) sites. For this analysis, we mapped the WGR reads of *P. vulgaris* to the chromosome‐scale reference genome of *P. veris* (Potente, Léveillé‐Bourret, et al., 2022) because it provides a better annotation of all *S*‐genes and their paralogs outside the *S*‐locus than the *P. vulgaris* reference genome. Prior to mapping, we annotated sites at 4‐ and 0‐fold degenerate sites as synonymous and non‐synonymous sites, respectively, for all nine genes using the script NewAnnotateRef.py (https://github.com/fabbyrob/science/tree/master/pileup_analyzers [last accessed on March 25, 2022]) (Williamson et al., 2014). Mapping of sequencing reads, variant calling, and filtering were performed with BWA‐mem v7.17 (Li & Durbin, 2009) and GATK v4.1.2.0 (McKenna et al., 2010) as described in Section 2.3. Subsequently, we split the VCF files of each of the nine genes into synonymous and non‐synonymous sites using BEDtools v2.29.2 (Quinlan & Hall, 2010). Using these VCF files, we estimated π_S_ and π_N_ with pixy v.1.0.0 (Korunes & Samuk, 2021). Specifically, we estimated π_S_ and π_N_ of *S*‐locus paralogs in heterostyles (i.e., pins and thrums combined) and homostyles, while the estimates of π_S_ and π_N_ for *S*‐genes included thrums (since pins lack the *S*‐locus) and homostyles. Finally, we estimated the strength of purifying selection in all *S*‐genes and their paralogs by calculating the ratio of nucleotide diversity at non‐synonymous vs. synonymous sites (π_N_/π_S_).

### 2.6 *S*‐locus genotypes in natural populations of *P. vulgaris*

To determine whether homostyles and thrums have either a haploid (i.e., S*/0 or *S*/0, respectively) or diploid (i.e., S*/S* or *S*/*S*, respectively) *S*‐locus, we calculated the relative sequencing depth of the *S*‐locus (*Rel*_*S*‐locus depth_), as follows. We first estimated the average site depth of the *S*‐locus coding regions (depth_*S*‐locus_) and of the genome‐wide coding regions (depth_Genome‐wide_) from filtered VCF files using BCFtools v1.9, as indicated in Section 2.5, then we calculated *Rel*_*S*‐locus depth_ as depth_*S*‐locus_/depth_Genome‐wide_. This normalization allowed us to account for differences of sequencing depth among individuals. A *Rel*_*S*‐locus depth_ value of approximately 0.5 ± 0.25 indicates a haploid *S*‐locus (S*/0 or *S*/0 for homostyles and thrums, respectively), while a value close to 1 ± 0.25 indicates a diploid *S*‐locus (S*/S* or *S*/*S* for homostyles and thrums, respectively).

To determine whether the proportions of individuals carrying 0/0‐, *S*/0‐, S*/S*‐, and S*/0‐genotypes in natural populations support the model with lower or the one with equal viability of S*/S*‐homostyles (Figure 1c,d), we compared the observed frequencies of the four genotypes in EN4‐T, EN5‐T, and EN6‐M with the expected genotype frequencies predicted by Crosby's model (Crosby, 1949; Figure 1c,d). In brief, the model estimates the proportion of pin (p), thrum (q), and the two types of homostylous (r and s) genotypes in a population at each generation using the proportion of p, q, r, and s individuals of the previous generation (Table S1). Specifically, p, q, r, and s at each generation are calculated by summing up the expected offspring proportion of pins, thrums, and homostyles following each possible cross in the population (see Table S1 for all possible crosses). All offspring is equally viable except for the S*/S*‐homostyles, whose viability can be either equal or 35% lower than that of the other genotypes (viability whose fitness is determined by the proportion of germinated seeds that develop into a seed‐producing plant; v in Table S1). Additionally, the original model assumed that pins have a selfing rate of 0.10 (Crosby, 1949). However, eliminating the assumption of selfing in pins does not change any of the results of the model (de Jong & Klinkhammer, 2005). Crosby's original model rested on the previous genetic model for the *S*‐locus, which assumed that thrums were typically heterozygous dominant at the *S*‐locus (i.e., *S*/*s*), pins homozygous recessive (i.e., *s*/*s*), and homostyles had one or two copies of a disrupted *S*‐locus (i.e., s*/*s* and s*/s*). The recently discovered hemizygosity of the *S*‐locus does not change any assumption of the simulation, which can be adjusted by assuming that *S*/*s*, *s*/*s*, s*/*s*, and s*/s* correspond to *S*/0, 0/0, S*/0, and S*/S*, respectively (Figures 1c,d).

Using Crosby's equations (1949), we first calculated the expected frequencies of all four genotypes (0/0‐, *S*/0‐, S*/S*‐, and S*/0) at generations 10, 20, 30, and 40 after the onset of homostyly, specifying either lower (*v* = 0.65) or equal (*v* = 1) viability of S*/S*‐homostyles. Since natural frequencies of the four genotypes might be compatible with levels of viability not included in Crosby's (1949) model, we additionally estimated expected genotype frequencies under *v* = 0.9, 0.8, 0.7, 0.6, and 0.5. Viability values below 0.5 were not used because phenotypic frequencies reflecting these conditions (roughly equal frequencies of pins and homostyles and absence of thrums) have never been reported in natural populations. Second, we estimated the observed frequencies of all four genotypes as follows. We tallied the raw counts of pins (0/0‐genotypes) and thrums (S/0‐genotypes) in EN4‐T and EN5‐T based on previous population surveys (T04 and T07, respectively, in Mora‐Carrera et al., 2021; Section 2.2, here). Furthermore, we calculated the number of homostyles with S*/0‐ and S*/S*‐genotypes in EN4‐T, EN5‐T, and EN6‐M by multiplying the proportion of S*/0‐ and S*/S*‐genotypes reported here (see Section 3.3) by the raw number of homostyles in each of the three populations. Finally, to determine whether observed and expected genotype frequencies are compatible with Crosby's model under lower or equal viability of S*/S*‐homostyles at 10, 20, 30, and 40 generations, we used chi‐squared tests with Bonferroni corrections. A significant difference between observed and expected frequencies indicates that observed genotype frequencies are not compatible with Crosby's model, while non‐significant results indicate that they are compatible with it.

## 3 RESULTS

### 3.1 Mutations putatively disrupting *CYP* ^*T*^ function in P. vulgaris

The use of newly generated WGR data enabled the identification of novel mutations in the exonic, intronic, and upstream regions of *CYP* ^*T*^ and exonic rearrangements in *CYP* ^*T*^ that could not be discovered by using the exon‐by‐exon Sanger sequencing approach employed in a previous study (Mora‐Carrera et al., 2021).

#### 3.1.1 Disruptive mutations in *CYP* ^*T*^ coding region

We discovered four, never‐before reported, non‐synonymous *CYP* ^*T*^ mutations in different exons of thrum individuals. Specifically, in the 37 thrums analyzed here, two non‐synonymous mutations were detected in exon 1 of four out of five thrums from the Slovakian population SK‐D; one non‐synonymous mutation was found in exon 3 of two thrums from the English population EN5‐T; and one non‐synonymous mutation was identified in exon 5 of one thrum from the Swiss population CH‐D. In contrast to the non‐synonymous mutations discovered in homostyles (see below), none of the above‐mentioned mutations found in thrums introduced a premature stop codon.

**FIGURE 2:**
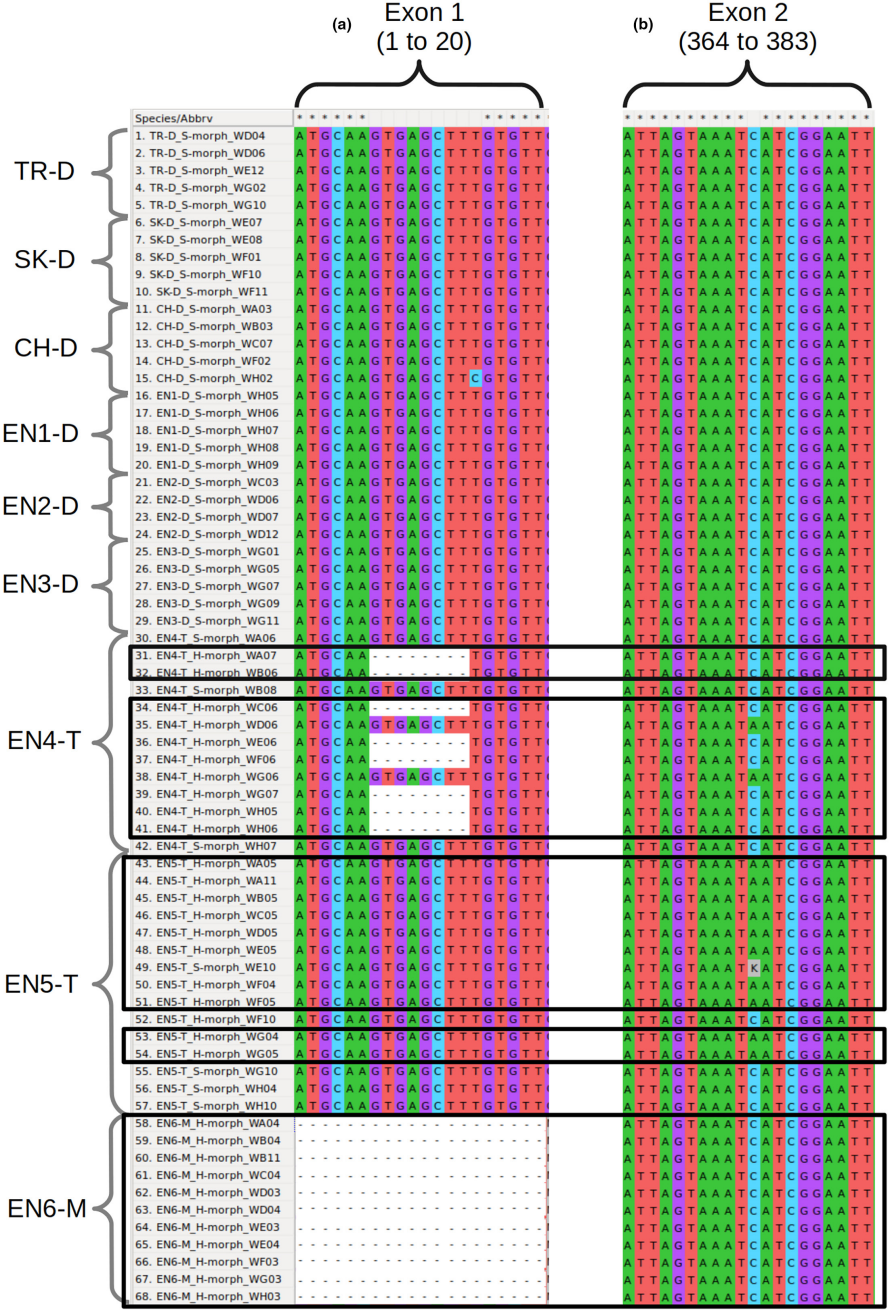
Alignment of *CYP* ^*T*^ exon 1 and 2 sequences containing homostyle‐specific mutations likely to disrupt *CYP* ^*T*^ function. No potential loss‐of‐function mutations were detected in exons 3, 4, 5, hence sequences of those exons are not shown. Sequences were extracted from whole genome resequencing (WGR) data of 37 thrums and 31 homostyles from nine populations of *Primula vulgaris* in Turkey (TR), Slovakia (SK), Switzerland (CH), and England (EN). (a) Sequence alignment of positions 1 to 20 of *CYP* ^*T*^ exon 1 (286 bp long). Eight homostyles of EN4‐T show an 8 bp‐deletion disrupting *CYP* ^*T*^ reading frame. No homostyles of EN6‐M showed sequencing reads mapping to exon 1, implying its loss (see also Figure S2). (b) Sequence alignment of positions 364 to 383 of *CYP* ^*T*^ exon 2 (227 bp long). A non‐synonymous mutation in position 374 that introduces an early stop codon occurs in 12 homostyles of EN4‐T and EN5‐T and one thrum of EN4‐T. This latter thrum is heterozygous and carries one functional and one disrupted copy of *CYP* ^*T*^. Homostyles are enclosed in black squares. The mutations shown in (a) and (b) correspond to the *CYP* ^*T*^‐6 and *CYP* ^*T*^‐2 alleles in Figure 1b, respectively. Population abbreviations as in Table 1.

As expected (Huu et al., 2016; Li et al., 2016; Potente, Léveillé‐Bourret, et al., 2022), we detected putatively disruptive *CYP* ^*T*^ mutations exclusively in homostyles (Figure 2), confirming previous results (Mora‐Carrera et al., 2021; Figure 2). Specifically, we corroborated a previously reported non‐synonymous mutation [Serine to Stop codon] in exon 2 (referred to as allele *CYP* ^*T*^‐2 in Mora‐Carrera et al., 2021) and an 8 bp deletion in exon 1 that shifts the open reading frame of *CYP* ^*T*^ (referred to as allele *CYP* ^*T*^‐6 in Mora‐Carrera et al., 2021; Figure 1b). Both previously reported mutations in *CYP* ^*T*^‐2 and *CYP* ^*T*^‐*6* introduce a premature stop codon, likely causing incomplete *CYP* ^*T*^ translation. In the present WGR dataset, *CYP* ^*T*^‐*2* occurred in two homostyles of population EN4‐T and all 10 homostyles of EN5‐T, while *CYP* ^*T*^‐*6* occurred in eight homostyles of EN4‐T.

#### 3.1.2 Mutations in intronic regions of *CYP* ^*T*^

Our analysis of the four *CYP* ^*T*^ introns in 37 thrums and 31 homostyles discovered a total of 1131 SNPs along a total intronic length of 66,515 bps. Most variant sites [1043 SNPs] were detected in non‐English populations (TR, CH, and SK; Figure S2). A total of 24 SNPs were present in homostyles, of which seven were exclusive to homostyles (i.e., absent from thrums; Table S2). However, only two out of the seven intronic homostyle‐specific SNPs segregated in more than one homostylous individual. Specifically, the intronic SNP at position 259,566 was shared by all eight individuals carrying *CYP* ^*T*^‐6, whereas the intronic SNP at position 275,096 was specific to all 11 individuals carrying *CYP* ^*T*^‐1 (Figure S1). Therefore, no intronic SNP was fixed in all 31 homostyles.

#### 3.1.3 Structural rearrangements in *CYP* ^*T*^

**FIGURE 3:**
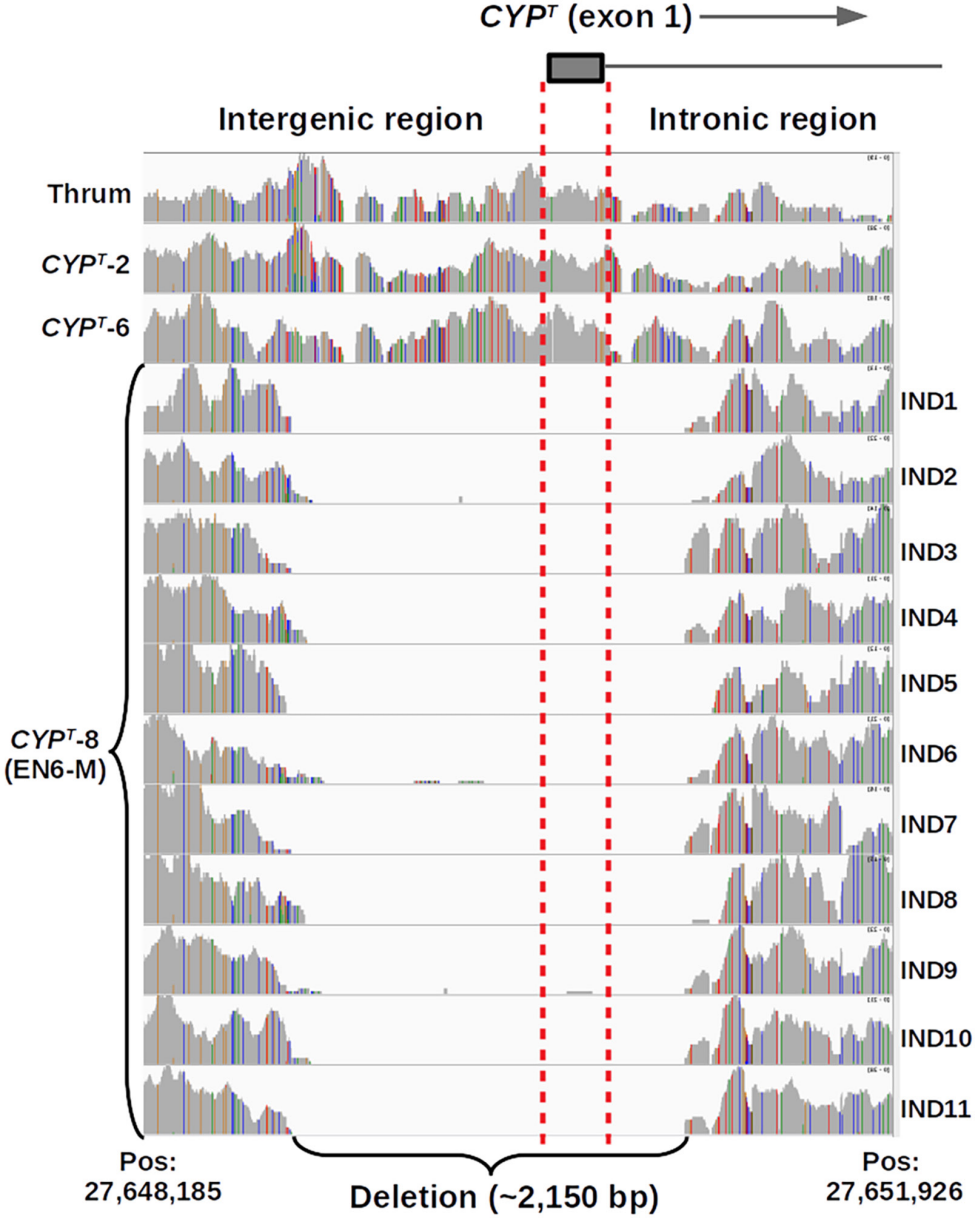
Integrative Genomic Viewer (IGV) plot showing sequencing reads mapping to exon 1 of *CYP* ^*T*^ and its flanking regions to the right (intronic region) and to the left (intergenic region) in one thrum individual, two homostyles with different *CYP* ^*T*^ alleles (see Figure 1), and the 11 homostylous individuals of the monomorphic population EN6‐M showing the absence of sequencing reads mapping to a ~2150 bp deletion encompassing exon 1 (286 bp long) of *CYP* ^*T*^. No other thrum or homostyle individuals showed this deletion.

A drop in sequencing coverage compared to the rest of the genome revealed for the first time a large deletion (ca. 2150 bps) encompassing *CYP* ^*T*^ exon 1 and its upstream and downstream regions (Figure 3). This 2150 bps deletion was detected exclusively in the 11 homostyles from population EN6‐M (Figures 2 and 3). Additional analyses of WGR data in IGV showed that a small number of reads within this region had paired mates mapping to different chromosomes (not shown), suggesting a local deletion of exon 1 and its potential translocation to another location in the genome. Notably, the remaining four *CYP* ^*T*^ exons in these homostyles showed sequencing‐read coverage values comparable to average sequencing‐read coverage across the genome and did not exhibit any additional disruptive mutations. Apart from the local deletion of exon 1, we did not identify additional inversions or translocations involving the remaining *CYP* ^*T*^ exons and introns. We designated this previously unreported allele as *CYP* ^*T*^‐8 (Figure 1b).

#### 3.1.4 Mutations in putative promoter region of *CYP* ^*T*^

We identified 20 conserved motifs in the region upstream of *CYP* ^*T*^ that are potential binding sites for seven different transcription‐factor families, as summarized in Table 2. Among these motifs, 10 were associated with various subfamilies of the DREB (Dehydration‐Responsive Element‐Binding protein) group within the ERF/AP2 transcription‐factor family, four were related to the MYB (Myeloblastosis viral oncogene) transcription factor, two were associated with the basic leucine‐zipper transcription factor, and one each were linked to the telomere‐repeat binding factor, basic pentacysteine, CHC (Clathrin Heavy Chain) protein, and a FAR1 (Fatty Acyl‐CoA Reductase 1)‐related protein. All these conserved motifs were found within a range of 1361–15 base pairs upstream of *CYP* ^*T*^ exon 1. Since *CYP* ^*T*^ is a brassinosteroid‐degrading enzyme expressed exclusively in the style in *P. vulgaris* (Huu et al., 2016), we focused on eight conserved motifs involved in brassinosteroid regulation and associated with genes expressed in the carpel of *Arabidopsis thaliana*, based on the TAIR database (Table 2). Inspection of the aligned 3 kb sequences upstream of *CYP* ^*T*^ in 20 out of the 31 homostyles indicated that such sequences were identical to those of the 37 thrums with a functional *S*‐locus (Appendix S1). However, in the 11 homostyles with the 2150 bp deletion affecting *CYP* ^*T*^ exon 1 and surrounding upstream and downstream regions (i.e., *CYP* ^*T*^‐8 allele: see above), all eight conserved motifs were absent from the promoter region. In summary, *CYP* ^*T*^ promoter regions are conserved and likely functional in all analyzed homostyles except for the 11 homostyles with a deleted *CYP* ^*T*^ exon 1 (*CYP* ^*T*^‐8 allele), which also appear to lack the promoter region.

**TABLE 2:** Summary of the 20 putative transcription factor (TF) binding sites upstream of *CYP* ^*T*^ in *Primula vulgaris* estimated from motif enrichment analyses (see Section 2).

TAIR ID CODE	TF FAMILY	Expressed in carpel	Expressed with BRs treatment	Distance from exon 1 *CYPT* (in bp)	Consensus sequence
AT1G01250	DREB	Yes	No	1361	TGTCGGTGRHKDNGD
AT1G19210	DREB	Yes	Yes	917	DTGGWCGGTGRHGR
AT1G36060	DREB	No	No	460	DTKGKCGGTGGHGR
AT4G28140	DREB	No	Yes	177	KGTCGGTGGHGRNGD
AT5G65130	DREB	No	Yes	177	HYNHHNHYDCCRCCGMCMW
AT3G50260	DREB	No	No	174	CCDYCDCCACCGMCA
AT5G67190	DREB	Yes	No	173	YCDYCDCCACCGACA
AT2G23340	DREB	Yes	Yes	173	HDWTGTCGGTGRHDNND
AT4G06746	DREB	No	–	173	HNNNNNHDYCACCGACAWH
AT1G21910	DREB	Yes	Yes	171	YCNCCDYCDYCDCCACCGMC
AT5G42520	BPC	Yes^a^	No	171	CTCTCTCTCTCTCTCTCTCTC
AT3G22780	CHC	Yes^a^	Yes	171	WWTTWAAAATTTAAA
AT3G23250	MYB	No	No	171	YHHHAHHWHHYYCACCAACCH
AT4G01680	MYB	No	Yes	168	WGGTWGGTRRRNNDD
AT1G09540	MYB	No	–	160	NYYYACCWACCWH
AT1G34670	MYB	No	No	80	NNWDBYYCACCWAMC
AT5G67580	TRBF2	Yes	Yes	64	WWWWHTWWRCCCTAAWTHH
AT4G38170	FAR1‐related	Yes^a^	Yes	64	CTCTCTCTCTCTCTCTCTCTC
AT2G40620	bZip	Yes	Yes	16	NMCAGCTGKCA
AT1G06850	bZip	Yes	Yes	16	DTGMCAGCTKGKHW

*Note*: Table includes “The *Arabidopsis* Information Resource” (TAIR) identification code, the TF family associated with the conserved motifs, whether or not the genes associated with this conserved motif are expressed in the carpel and in the presence of brassinosteroids (BR) in *Arabidopsis thaliana* based on the TAIR database, approximate upstream distance from the first exon of *CYP* ^*T*^, and consensus motif sequence of the TF binding site.

Abbreviations: BPC, basic pentacysteine; bZIP, basic leucine zipper transcription factor; CHC, Chlathrin heavy chain; DREB, dehydration‐responsive element‐binding; FAR1, Fatty Acyl‐CoA Reductase 1; MYB, myeloblastosis viral oncogene; TRBF2, Telomore Repeat Binding Factor 2.

^a^ Highly overexpressed.

Our analysis of the putative cis‐regulatory elements upstream of *CYP* ^*T*^ indicates that both *P. vulgaris* and *P. veris* have similar cis‐elements (Figure S2), as expected in closely related species. Further examination of these elements in the 37 thrums and 31 homostyles of *P. vulgaris*, and one individual of *P. veris* indicates that they are present in the homostyles and that their sequence is identical to that of the thrums, implying that the putative *cis*‐regulatory elements identified here are still functional in the homostyles.

### 3.2 Genetic variation in *S*‐genes *and their paralogs*

Our results showed that, as expected due to *S*‐locus hemizygosity, π_S_ was lower in *S*‐genes of thrums (0.0012 ± 0.0006 [mean ± SE]) than in their paralogs in pins and thrums (0.0034 ± 0.0008; Table 3A). Moreover, π_S_ in homostyles was zero for both *S*‐genes and their paralogs, except for *GLO* ^*T*^ and *CYP734A51*, where π_S_ was extremely low (0.0012 and 0.0008, respectively; Table 3B), supporting the prediction that the shift to predominant selfing should be associated with lower π_S_ in homostyles than in heterostyles.

**TABLE 3:** Estimates of nucleotide diversity at synonymous (*n*_S_), non‐synonymous sites (*n*_N_), and the strength of purifying selection (nN/nS) of all five *S*‐genes (*CYP* ^*T*^, *GLO* ^*T*^, *CCM* ^*T*^, *KFB* ^*T*^, and *PUM* ^*T*^) and their paralogs (*CYP75341*, *GLO1*, *CCM1*, and *KFB1*) in (A) thrums (S‐locus genes) and heterostyles (paralogs) and (B) homostyles of *Primula vulgaris*.

Gene	n	π_S_	π_N_	π_N_/π_S_
(A)				
*CYP* ^*T*^	37^a^	0.0026	0.0007	0.28
*GLO* ^*T*^	37^a^	0	0.0015	NA
*CCM* ^*T*^	37^a^	0.0018	0.0016	0.91
*KFB* ^*T*^	37^a^	0.0004	0.0007	1.83
*PUM* ^*T*^	37^a^	0.0001	0.0011	10.36
	Average (±SE)	0.0010 (±0.0005)	0.0011 (±0.0002)	3.35 (±2.04)
		0.0012 (±0.0006)^c^	0.0011 (±0.0003)^c^	1.01 (±0.37)^c^
*CYP734A51*	74^b^	0.0040	0.0015	0.38
*GLO1*	74^b^	0.0019	0	NA
*CCM1*	74^b^	0.0019	0.0029	1.50
*KFB1*	74^b^	0.0056	0.0013	0.23
	Average (±SE)	0.0034 (±0.0008)	0.0014 (±0.005)	0.53 (±0.29)
(B)				
*CYP* ^*T*^	31	0	0.0005	NA
*GLO* ^*T*^	31	0.0012	0	0
*CCM* ^*T*^	31	0	0	0
*KFB* ^*T*^	31	0	0	0
*PUM* ^*T*^	31	0	0	0
	Average (±SE)	0.0002 (±0.00)	0.0001 (±0.00)	0
*CYP734A51*	31	0.0008	0.0002	0.28
*GLO1*	31	0	0	0
*CCM1*	31	0	0.0003	NA
*KFB1*	31	0	0.0008	NA
	Average (±SE)	0.0002 (±0.00)	0.0003 (±0.0001)	0.14 (±0.14)

*Note*: *n*, sample size.

Abbreviation: NA, not available.

^a^ Thrums.

^b^ Pins and thrums.

^c^ Average without *PUM* ^*T*^.

Furthermore, our results indicated that, on average, π_N_/π_S_ values were higher for *S*‐genes of thrums than for their paralogs in pins and thrums (1.01 ± 0.37 vs. 0.53 ± 0.29, respectively; Table 3A), implying lower purifying selection in *S*‐genes. Additionally, π_N_/π_S_ was lower in *KFB1* than in *KFB* ^*T*^ (π_N_/π_S_ = 0.23 and 1.83, respectively), but higher in *CCM1* than in *CCM* ^*T*^ (π_N_/π_S_ = 1.5 and 0.91, respectively; Table 3A), implying stronger and weaker purifying selection on the two paralogs than on their respective *S*‐genes, respectively. Finally, we found that, within the *S*‐locus, π_N_/π_S_ was higher for *CCM* ^*T*^, *KFB* ^*T*^, and *PUM* ^*T*^ (π_N_/π_S_ = 0.91, 1.83, and 10.36, respectively; Table 3B) than for *CYP* ^*T*^ (π_N_/π_S_ = 0.28), whereas π_N_/π_S_ in *GLO* ^*T*^ was not calculated due to the lack of variation at synonymous sites in this gene. In homostyles, π_N_/π_S_ was zero for most *S*‐genes and their paralogs, except for *CYP734A51*, due to the lack variation at synonymous and non‐synonymous sites (Table 3B *)*.

### 3.3 *S*‐locus genotypes in natural populations of *P. vulgaris*

**FIGURE 4:**
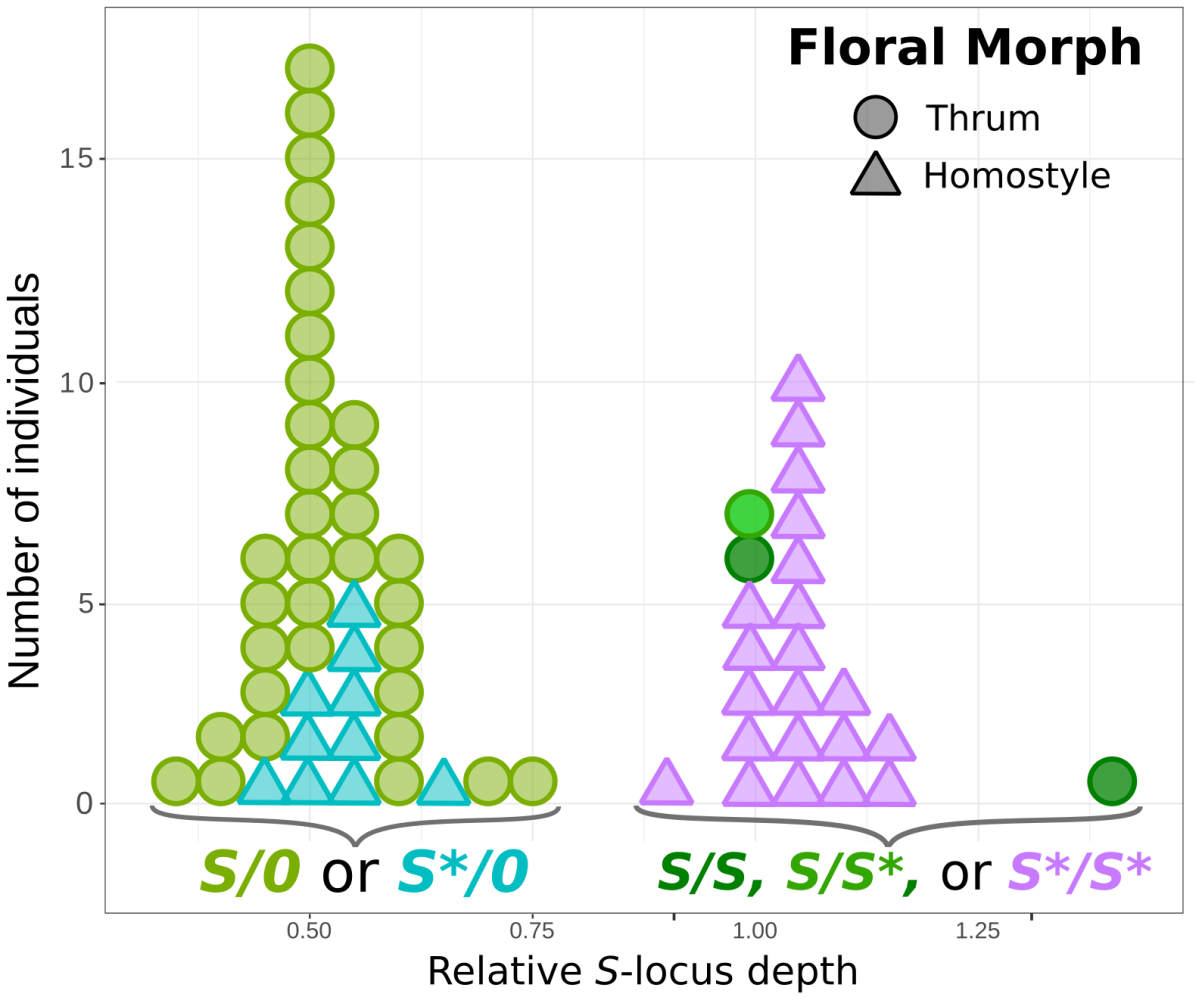
Number of individuals with either haploid (*S*/0 = 34; S*/0 = 10) or diploid (S/S = 2; S/S* = 1; S*/S* = 21) *S*‐locus, as inferred from the relative depth of sequencing reads mapping to the *S*‐locus (see Section 2). Thrums are represented as circles (*n* = 37) and homostyles as triangles (*n* = 31). The same colors used in Figure 1 were also used here to represent the different genotypes of the floral morphs: Light green = *S*/0; medium green = *S*/S*; dark green = S/S; turquoise = S*/0; purple = S*/S*. A total of 68 individuals were sampled from dimorphic (i.e., only pins and thrums), trimorphic (i.e., pins, thrums, and homostyles) and monomorphic (i.e., only homostyles) populations of *P. vulgaris* in TR, SK, CH, and EN (Table 1).

Relative *S*‐locus sequencing depth (*Rel*_*S*‐*locus depth*_) allowed us to determine *S*‐locus ploidy in the analyzed thrums and homostyles. Of the 37 thrums collected from six dimorphic (i.e., pins and thrums) and two trimorphic (i.e., pins, thrums, and homostyles) populations across our sampling range, 34 had a haploid *S*‐locus (i.e., S/0) and three had a diploid *S*‐locus (*S*/*S*; Figure 4). Of these three thrums, two were homozygous for the functional copy of *CYP* ^*T*^ (*CYP* ^*T*^‐*1*/*CYP* ^*T*^‐*1*; i.e., *S*/*S*) and belonged to one trimorphic and one dimorphic population each, respectively (Table 1), while one thrum was heterozygous and carried one functional and one disrupted copy of *CYP* ^*T*^ (*CYP* ^*T*^‐*1*/*CYP* ^*T*^‐*2*; i.e., *S*/S*). Of the 31 homostyles, 10 (32%) had a haploid *S*‐locus (S*/0) and 21 (68%) had a diploid *S*‐locus (S*/S*) (Figure 4). Specifically, S*/0‐homostyles represented 40% and 60% of homostyles in the trimorphic populations EN4‐T and EN5‐T, respectively, while all tested homostyles of the monomorphic population EN6‐M had the S*/S*‐genotype (Table 1; purple triangles in Figure 4).

We also calculated expected and observed frequencies of 0/0, S/0‐, S*/0‐, and S*/S*‐genotypes under different assumptions for viability of S*/S*‐genotypes compared with the other three genotypes and after different numbers of generations following the origin of homostyles (Table 4). The results of chi‐squared tests showed that non‐significant differences were found only in seven cases, of which four occurred in the monomorphic, homostylous population EN6‐M and three in the two trimorphic populations EN5‐T and EN4‐T. Specifically, in EN6‐M, observed frequencies matched expected frequencies at generations 30 and 40 under the assumption of equal and slightly lower viability (*v* = 1 and 0.9, respectively) for the S*/S*‐homostyles. In EN5‐T, observed frequencies matched expected frequencies at 20 generations under lower viability (*v* = 0.8) of S*/S*‐homostyles. Finally, in EN4‐T, observed frequencies matched expected genotypic frequencies at generations 20 and 30 under levels of viability for S*/S*‐homostyles close or equal to those of the model in Figure 1c (i.e., *v* = 0.70 and 0.65, respectively).

**TABLE 4:** Comparison of expected frequencies and observed frequencies of *S*‐locus genotypes (0/0‐, *S*/0‐, S*/0‐, and S*/S*) in two trimorphic (EN4‐T and EN5‐T) and one monomorphic populations (EN6‐M).

(A)		Observed frequencies (0/0:S/0:S*/0:S*/S*)		
Generation	*v* = 1	EN4‐T	EN5‐T	EN6‐M
	Expected freq (0/0:S/0:S*/0:S*/S*)			
10	0.50:0.42:0.05:0.03	0.36:0.16:0.29:0.19**	0.29:0.09:0.25:0.36**	0:0:0:1**
20	0.17:0.04:0.29:0.50	0.36:0.16:0.29:0.19**	0.29:0.09:0.25:0.36**	0:0:0:1**
30	0.02:0.00:0.06:0.92	0.36:0.16:0.29:0.19**	0.29:0.09:0.25:0.36**	**0:0:0:1** ^**NS**^
40	0.00:0.00:0.01:0.99	0.36:0.16:0.29:0.19**	0.29:0.09:0.25:0.36**	**0:0:0:1** ^**NS**^

(B)		Observed frequencies (0/0:S/0:S*/0:S*/S*)		
Generation	*v* = 0.9	EN4‐T	EN5‐T	EN6‐M
	expected freq (0/0:S/0:S*/0:S*/S*)			
10	0.46:0.34:0.14:0.07	0.36:0.16:0.29:0.19**	0.29:0.09:0.25:0.36**	0:0:0:1**
20	0.15:0.02:0.29:0.54	0.36:0.16:0.29:0.19**	0.29:0.09:0.25:0.36**	0:0:0:1**
30	0.04:0.00:0.11:0.85	0.36:0.16:0.29:0.19**	0.29:0.09:0.25:0.36**	**0:0:0:1** ^**NS**^
40	0.01:0.00:0.04:0.95	0.36:0.16:0.29:0.19**	0.29:0.09:0.25:0.36**	**0:0:0:1** ^**NS**^

(C)		Observed frequencies (0/0:S/0:S*/0:S*/S*)		
Generation	*v* = 0.8	EN4‐T	EN5‐T	EN6‐M
	Expected freq (0/0:S/0:S*/0:S*/S*)			
10	0.48:0.48:0.10:0.04	0.36:0.16:0.29:0.19**	0.29:0.09:0.25:0.36**	0:0:0:1**
20	0.25:0.05:0.37:0.33	0.36:0.16:0.29:0.19**	**0.29:0.09:0.25:0.36** ^**NS**^	0:0:0:1**
30	0.12:0.00:0.27:0.62	0.36:0.16:0.29:0.19**	0.29:0.09:0.25:0.36**	0:0:0:1**
40	0.08:0.00:0.19:0.73	0.36:0.16:0.29:0.19**	0.29:0.09:0.25:0.36**	0:0:0:1**

(D)		Observed frequencies (0/0:S/0:S*/0:S*/S*)		
Generation	*v* = 0.7	EN4‐T	EN5‐T	EN6‐M
	Expected freq (0/0:S /0:S*/0:S*/S*)			
10	0.50:0.40:0.07:0.03	0.36:0.16:0.29:0.19**	0.29:0.09:0.25:0.36**	0:0:0:1**
20	0.35:0.13:0.35:0.17	**0.36:0.16:0.29:0.19** ^**NS**^	0.29:0.09:0.25:0.36**	0:0:0:1**
30	0.21:0.01:0.40:0.38	0.36:0.16:0.29:0.19**	0.29:0.09:0.25:0.36**	0:0:0:1**
40	0.17:0.00:0.37:0.46	0.36:0.16:0.29:0.19**	0.29:0.09:0.25:0.36**	0:0:0:1**

(E)		Observed frequencies (0/0:S/0:S*/0:S*/S*)		
Generation	*v* = 0.65	EN4‐T	EN5‐T	EN6‐M
	Expected freq (0/0:S /0:S*/0:S*/S*)			
10	0.52:0.46:0.01:0.01	0.36:0.16:0.29:0.19**	0.29:0.09:0.25:0.36**	0:0:0:1**
20	0.48:0.37:0.12:0.03	0.36:0.16:0.29:0.19**	0.29:0.09:0.25:0.36**	0:0:0:1**
30	0.34:0.01:0.38:0.18	**0.36:0.16:0.29:0.19** ^**NS**^	0.29:0.09:0.25:0.36**	0:0:0:1**
40	0.24:0.01:0.43:0.32	0.36:0.16:0.29:0.19**	0.29:0.09:0.25:0.36**	0:0:0:1**

*Note*: Expected frequencies of *S*‐locus genotypes were estimated at 10, 20, 30, and 40 generations after the onset of homostyly with different levels of viability (*v*) for S*/S*‐homostyles relative to S*/0‐homostyles, *S*/0‐thrums, and 0/0‐pins: (A) *v* = 1 (see Figure 1d), (B) *v* = 0.9, (C) *v* = 0.8, (D) *v* = 0.7, and (E) *v* = 0.65 (see Figure 1c). Significance was estimated using chi‐square tests with Bonferroni corrections. Non‐significant differences between expected frequencies and observed frequencies of *S*‐locus genotypes (boldfaced) indicate the conditions under which observed genotypic frequencies are compatible with Crosby's model predictions (Crosby, 1949).

** = *p* < .01; ^NS^, non‐significant differences.

## 4 DISCUSSION

We integrated whole‐genome resequencing data from a comprehensive sampling of long‐monitored, English populations and from other European populations of *P. vulgaris* with knowledge of the recently assembled genomes of *P. vulgaris* and *P. veris* to explain the causes and consequences of the transition from distyly to homostyly. We identified a novel loss‐of‐function structural rearrangement in *CYP* ^*T*^ associated with the transition to homostyly that had remained undetected using exonic Sanger sequencing (Mora‐Carrera et al., 2021). Importantly, we found no evidence for a potential single origin of homostyly in *P. vulgaris* via mutations in the *CYP* ^*T*^ promoter, *cis*‐regulatory, and intronic regions, or structural mutations involving *CYP* ^*T*^ exons, thus the previously supported hypothesis of multiple transitions to homostyly via independent loss‐of‐function mutations in *CYP* ^*T*^ exons stands (Mora‐Carrera et al., 2021). Furthermore, population genetic analyses validated theoretical expectations of decreased genetic diversity in *S*‐genes due to hemizygosity. However, contrary to expectations, purifying selection was not consistently lower in all *S*‐genes than in their paralogs. Finally, the genomic resources newly available in the *Primula* system enabled, for the first time, the testing of long‐standing predictions on changing frequencies of *S*‐locus genotypes during intraspecific transitions from distyly to homostyly, partially supporting the possible role of viability (i.e., seed germination and successful development into seed‐bearing plants) differences between homostyles with haploid vs. diploid *S*‐locus genotypes in preventing the fixation of homostyly. Altogether, our study provides a detailed overview of the early molecular and population genetic causes and consequences of mating‐system transitions.

### 4.1 Genetic basis of transitions from distyly to homostyly in *Primula vulgaris*

Shifts from outcrossing to selfing are common in flowering plants and can be caused by loss‐of‐function mutations in the genes of interest, structural rearrangements of their exons, or mutations in their promoters (Shimizu & Tsuchimatsu, 2015). One question concerns whether disruptive mutations in the alleles that determine outcrossing act dominantly or recessively. In Brassicaceae, loss of self‐incompatibility often stems from mutations in dominant alleles of genes controlling this trait (Bachmann et al., 2019; Busch et al., 2011; Nasrallah, 2017; Tsuchimatsu et al., 2012), although in *Arabidopsis lyrata* loss of self‐incompatibility caused by mutations in recessive alleles has also been discovered (Mable et al., 2017). In *Primula*, the *S*‐locus controlling distyly is hemizygous in both *P. vulgaris* and *P. veris* (Huu et al., 2016, 2020), but previous models assumed *S*‐locus heterozygosity, with thrum phenotype associated with the dominant *S*‐locus allele (Bateson & Gregory, 1905). Based on this model and greenhouse crossing experiments, Crosby (1949) assumed that *S*‐locus alleles associated with homostyly should be recessive. Previously, we documented seven *CYP* ^*T*^ haplotypes (*CYP* ^*T*^‐2 to *CYP* ^*T*^‐7) with putative loss‐of‐function mutations that occur exclusively in *P. vulgaris* homostyles (Mora‐Carrera et al., 2021). Two of these haplotypes (*CYP* ^*T*^‐2 and *CYP* ^*T*^‐6) have an early stop codon causing premature termination of translation. Our results indicate that, whether hemizygous or homozygous, these disrupted *CYP* ^*T*^ alleles lead to homostyly. However, *CYP* ^*T*^‐2 behaves recessively in the single heterozygous individual carrying one functional and one disrupted copy of *CYP* ^*T*^ (i.e., *CYP* ^*T*^‐1/*CYP* ^*T*^‐2, represented as *S*/S* in Figure 4), thus determining a thrum phenotype. This finding aligns with results of crossing experiments between thrums and homostyles of *Primula oreodoxa* showing that S* is recessive to *S* when the two alleles co‐occur (Yuan et al., 2018), corroborating Crosby's prediction (1949). Therefore, our results indicate that the presence of a disrupted *CYP* ^*T*^ allele does not alter the thrum morph when paired with a functional *CYP* ^*T*^ allele, hence the disrupted allele acts recessively.

In addition to loss‐of‐function mutations in coding regions, mating‐system shifts can also stem from transcription silencing or exonic rearrangements in pertinent genes (Chakraborty et al., 2023). For instance, down‐regulation caused by transposon‐like insertions in the promoter regions of the male‐determining self‐incompatibility genes *MGST* and *BnSP11*‐*1* trigger the shift from self‐incompatibility to self‐compatibility in *Prunus avium* and *Brassica napus*, respectively (Gao et al., 2016; Ono et al., 2018). In *Primula vulgaris*, previous Sanger sequencing of individual *CYP* ^*T*^ exons identified homostyles with an apparently functional *CYP* ^*T*^ allele (*CYP* ^*T*^‐1; Figure 1b), suggesting that homostyles might also arise through *CYP* ^*T*^ silencing caused by a disruptive mutation in its promoter or intronic regions or via exonic rearrangements in *CYP* ^*T*^ not detectable via Sanger sequencing (Mora‐Carrera et al., 2021). However, 20 of the 31 homostyles analyzed in the present study did not share any homostyle‐specific SNP in the promoter or intronic region that was absent in the 37 thrums, implying that the shift to homostyly in these plants was likely caused by loss‐of‐function mutations in *CYP* ^*T*^ exons rather than in its promoter or intronic region. The remaining 11 homostyles were characterized by a large 2150 bp deletion that eliminated both *CYP* ^*T*^ exon 1 and its promoter region (Figure 3). Thus, our current results do not support the conclusion that mutations in the promoter region or exonic rearrangements in *CYP* ^*T*^ can alone cause the shift to homostyly in *P. vulgaris*.

The evidence above also has implications for determining whether homostyly arose once or multiple times in *P. vulgaris*. The single origin of homostyly, followed by independent mutations in *CYP* ^*T*^, would have been supported if all studied homostyles had shared the same promoter mutation or rearrangement in *CYP* ^*T*^. However, this is not the case, favoring the hypothesis of multiple origins of homostyly via independent mutations in *CYP* ^*T*^ exons, as previously proposed (Mora‐Carrera et al., 2021). Nevertheless, a study of a single homostyle from Chiltern Hills, England (population not included in our analyses), found reduced expression of *CYP* ^*T*^ when compared to a thrum, suggesting that epigenetic silencing might play a role in the shift to homostyly (Huu et al., 2016). The mentioned study however did not provide sequences of *CYP* ^*T*^ exons, thus it remains unknown whether they contained any potentially disruptive mutations in the coding region. Therefore, transcriptome analyses of homostylous flowers are necessary to conclusively discard the possibility that disruptive promoter mutations causing reduced *CYP* ^*T*^ expression might also cause the shift to homostyly.

Finally, it remains to be explained why the three homostyles previously thought to have the functional *CYP* ^*T*^‐1 allele based on Sanger sequencing of the five *CYP* ^*T*^ exons (Mora‐Carrera et al., 2021) were here found to contain the 2150 bp deletion including exon 1 (i.e., *CYP* ^*T*^ ‐8 haplotype: see Figures 1, 2, 3). A possible explanation is that exon 1 was deleted from the *S*‐locus (causing *CYP* ^*T*^ loss of function, hence homostyly) and translocated to a highly repetitive genomic region. The translocation could have allowed targeted amplification and subsequent Sanger sequencing using exon‐1‐specific PCR primers, while preventing exon‐1 detection via next generation sequencing due to biases arising, for example, during genomic DNA sonication used to produce short DNA fragments prior to short‐read library preparation (Garafutdinov et al., 2016; Jennings et al., 2017; Poptsova et al., 2014). Notably, a few low‐quality sequencing reads did map to *CYP* ^*T*^ exon 1, suggesting this exon is indeed present in the genome of these homostyles but was not successfully sequenced using short‐read sequencing methodology. Long‐read sequencing technologies capable of sequencing through repetitive regions would be necessary to definitively resolve whether *CYP* ^*T*^ exon 1 was translocated to a highly repetitive genomic region in these homostyles. To summarize, our findings indicate that the homostyles previously identified as having a functional *CYP* ^*T*^ allele in fact do possess a disrupted *CYP* ^*T*^ allele due to exon 1 deletion from the *S*‐locus (designated *CYP* ^*T*^‐8 allele: Figure 3). Overall, these results emphasize that not only non‐synonymous mutations or small deletions, but also structural rearrangements such as large deletions and translocations can cause mating‐system transitions.

### 4.2 Population genetic consequences of hemizygosity and transition to homosyly on *S*‐genes

One of the most notable, recent discoveries on the *S*‐locus is that it is hemizygous and present only in thrums in all systems where its genetic architecture has been investigated, including *Primula* (Huu et al., 2016; Li et al., 2016), *Turnera* (Shore et al., 2019), *Fagopyrum* (Fawcett et al., 2023; Matsui & Yasui, 2020), *Linum* (Gutiérrez‐Valencia et al., 2022), and *Gelsemium* (Zhao et al., 2023), representing different families and orders of flowering plants. The hemizygosity of the *S*‐locus should affect patterns of molecular diversity. Specifically, tight genetic linkage provided by recombination suppression in *S*‐genes and the fact that the *S*‐locus is present only in thrum individuals are expected to cause a reduction of *N_e_* and consequently a decrease of genetic diversity inside the *S*‐locus compared to other genomic regions (Gutiérrez‐Valencia et al., 2021). Our results demonstrate that the mean π_S_ of *S*‐genes (*CCM* ^*T*^, *GLO* ^*T*^, *CYP* ^*T*^, and *KFB* ^*T*^; π_S_: 0.0012 ± 0.0006) is lower than that of their paralogs (*CCM1*, *GLO1*, *CYP734A51*, and *KFB1*; π_S_: 0.0034 ± 0.0007) located elsewhere in the genome (Table 3). This result corroborates previous studies that found an overall decrease in genetic diversity between the *S*‐locus and its flanking regions in *Primula* (Potente, Léveillé‐Bourret, et al., 2022), *Linum* (Gutiérrez‐Valencia et al., 2022), and *Turnera* (Henning et al., 2023). Thus, our work confirms predictions that *S*‐locus genomic architecture influences patterns of molecular evolution in *S*‐genes.

The hemizygous, non‐recombining nature of the *S*‐locus also affects its response to natural selection when compared to recombining regions. Specifically, the increased linkage disequilibrium caused by the absence of recombination should lead to a reduction of *N_e_* within the *S*‐locus. This reduction of *N_e_* should, in turn, lead to less efficient purifying selection (Gossmann et al., 2011) on *S*‐genes compared to their paralogs outside the *S*‐locus. Consequently, increased degeneration due to accumulation of deleterious mutations is expected in these genes (Charlesworth & Charlesworth, 2000; Huu et al., 2016). Conversely, if selection to maintain function were strong, purifying selection should be more efficient on *S*‐genes than on their paralogs due to the dominant nature of the hemizygous *S*‐locus (Gutiérrez‐Valencia et al., 2021; Potente, Léveillé‐Bourret, et al., 2022). Regarding the former hypothesis, a greater accumulation of transposable elements in *S*‐locus non‐coding regions compared to the rest of the genome was detected, supporting the conclusion that purifying selection on the *S*‐locus might be relaxed (Potente, Léveillé‐Bourret, et al., 2022). However, whether the efficacy of purifying selection differs between coding regions of *S*‐genes and their paralogs remains poorly understood (Potente, Léveillé‐Bourret, et al., 2022). Our results indicate that, on average, *S*‐genes exhibit higher accumulation of non‐synonymous mutations than their paralogs, implying purifying selection is less effective on the former (π_N_/π_S_ = 1.01 ± 0.37 and 0.53 ± 0.25, respectively; Table 3), conformant with predicted effects of reduced *S*‐locus *Ne*. However, patterns of selective constraints within and outside the *S*‐locus vary among gene duplicates. For example, the strength of purifying selection is similar between *CYP* ^*T*^ and *CYP734A51*, albeit slightly stronger in the former (π_N_/π_S_ = 0.28 and 0.38, respectively). Conversely, purifying selection is less efficient in the *S*‐locus for KFB (π_N_/π_S_ = 1.83 [*KFB* ^*T*^] and 0.23 [*KFB1*]), whereas *CCM* shows the opposite pattern (π_N_/π_S_ = 0.91 [*CCM* ^*T*^] and 1.50 [*CCM1*]; Table 3). Taken together, the results imply that the effects of hemizygosity on purifying selection vary among *P. vulgaris S*‐genes, corroborating previous results in *P. veris* (Potente, Léveillé‐Bourret, et al., 2022).

A key question for the genetics of distyly concerns whether the strength and nature of selection differ among the genes within *S*‐locus. Among the three, nine, and five protein‐coding genes identified in the *S*‐locus of *Gelsemium*, *Linum*, and *Primula*, respectively, (Gutiérrez‐Valencia et al., 2022; Li et al., 2016; Potente, Léveillé‐Bourret, et al., 2022; Zhao et al., 2023) only two, namely *CYP* ^*T*^ and *GLO* ^*T*^ of *Primula*, have been functionally characterized, showing that *CYP* ^*T*^ determines short styles and female self‐incompatibility (Huu et al., 2016, 2022), while *GLO* ^*T*^ determines high anthers in thrums (Huu et al., 2020). However, it remains unclear whether *CCM* ^*T*^, *KFB* ^*T*^, and *PUM* ^*T*^ play a role in *Primula* distyly. The markedly reduced and non‐floral specific expression of *CCM* ^*T*^, *KFB* ^*T*^, and *PUM* ^*T*^ compared to *CYP* ^*T*^ and *GLO* ^*T*^ in both *P. vulgaris* and *P. veris* (Cocker et al., 2018; Potente, Stubbs, et al., 2022) cast doubt on whether the former three genes are essential for distyly. In the distylous *Gelsemium elegans* (Gentianales), the homolog of *Primula CCM* ^*T*^ was absent from the genome, while homologs of *KFB* ^*T*^ and *PUM* ^*T*^ were present but did not localize to the putative *S*‐locus and were expressed in both pin and thrum flowers (Zhao et al., 2023). Taken together, previous evidence suggests that *CCM* ^*T*^, *KFB* ^*T*^, and *PUM* ^*T*^ may not be essential for the core traits of distyly (i.e., reciprocal placement of sexual organs and self‐incompatibility); hence, they might be under relaxed purifying selection. If this is true, one might expect thrums to exhibit higher accumulation of non‐synonymous mutations in *CCM* ^*T*^, *KFB* ^*T*^, and *PUM* ^*T*^ than in *CYP* ^*T*^ and *GLO* ^*T*^. Our results support this prediction, for we found that, within the *S*‐locus, *CCM* ^*T*^, *KFB* ^*T*^, and *PUM* ^*T*^ (π_N_/π_S_ = 0.91, 1.83, and 10.36, respectively) compared to *CYP* ^*T*^ (π_N_/π_S_ = 0.28; Table 3A). It is unlikely that the results are explained by positive directional selection on advantageous non‐synonymous mutations of the three genes above in thrums, because positive selection should cause rapid fixation of advantageous mutations, hence the absence of polymorphism at non‐synonymous sites (Hahn, 2018), which is not what we found (Table 3A). To summarize, in *P. vulgaris* purifying selection seems stronger on the only two *S*‐genes for which a key function in distyly has been demonstrated (namely, *CYP* ^*T*^ and *GLO* ^*T*^) than on *CCM* ^*T*^, *KFB* ^*T*^, and *PUM* ^*T*^. Discovering whether the later three genes above may play a role in controlling ancillary traits of distyly (e.g., pollen size and number, and male incompatibility) requires additional functional studies in *Primula* and other distylous taxa.

Comprehensive population genetic analyses of variability in *S*‐genes and their paralogs had never been performed until now, due to missing knowledge of relevant genes, unavailability of sequences from said genes, and inadequate population sampling across extensive geographic ranges. Here, we expanded on previous Sanger sequencing analyses of *CYP* ^*T*^ in Somerset (England) populations (Mora‐Carrera et al., 2021) by analyzing also sequences of *S*‐genes and their paralogs extracted from WGR data of Slovakian, Swiss, and Turkish populations of *P. vulgaris*. First, homostyles, found exclusively in three Somerset populations, exhibited lower genetic diversity than thrums for both *S*‐genes and their paralogs (Table 3), corroborating previous reports of reduced genetic diversity in homostyles (Husband & Barrett, 1993; Ness et al., 2010; Yuan et al., 2017; Zhong et al., 2019; Zhou et al., 2017). Second, both *S*‐genes and their paralogs have markedly lower genetic variation in English populations than in other Eurasian populations of *P. vulgaris* (Table S3). This finding suggests a recent genetic bottleneck in English populations. This bottleneck could be associated with colonization of England following glacial retreat during the Last Glacial Maximum (ca. 10,000–12,000 years ago), as suggested for other plant species (Birks, 1989). Future genomic and demographic investigations will determine whether the signatures of genetic bottlenecks detected in *S*‐genes and their paralogs apply to the entire genome, thus helping to infer the timing and mode of *P. vulgaris* colonization of the British Isles.

### 4.3 Does lower viability of S*/S*‐homostyles prevent the fixation of homostyly in *P. vulgaris*?

Theoretical and experimental work suggests that, in the absence of inbreeding depression and assuming that all individuals produce equal number of seeds, once selfing originates, the selfing phenotype should increase in frequency and eventually become fixed over time (Charlesworth et al., 1990; Fisher, 1941; Lande & Schemske, 1985). In the transition from distyly to homostyly, Crosby's model (Crosby, 1949) predicted that the rate of increase and ultimate fixation of homostyles in a population depends on whether homostyles with diploid *S*‐locus have lower or equal viability as the other genotypes in the population (Figure 1c,d). The assumption of lower viability for S*/S*‐homostyles of *P. vulgaris* expanded upon prior evidence from crossing experiments in *P. sinensis* suggesting that homozygous dominant thrums had 30% lower viability than heterozygous thrums (Mather & Winton, 1941). More recently, results of crossing experiments in a *Primula* hybrid (*Primula* × *tommasinii*) were interpreted as evidence of inviability for *S*/*S*‐thrums (Kurian & Richards, 1997). Furthermore, population surveys of pin‐to‐thrum ratios in *P. oreodoxa* indicated that thrums were overrepresented at the seed (~1:3) but not adult stage (~1:1), implying that differences in viability could occur during the life cycle (Yuan et al., 2018). However, genotyping of thrums was not carried out, thus preventing the determination of whether the decrease of thrums from seed to adult stage was caused by lower viability of *S*/*S*‐thrums. Our observed frequencies of S*/0‐ and S*/S*‐homostyles from the two trimorphic (i.e., pins, thrums, and homostyles), English populations EN4‐T and EN5‐T of *P. vulgaris* are consistent with Crosby's prediction of a recent transition to homostyly (20–30 generations) under 30%–40% lower viability of S*/S*‐homostyles (Table 4), supporting the model that assumes lower fitness for S*/S*‐homostyles than S*/0‐homostyles (Figure 1c).

Conversely, the occurrence of a monomorphic, homostylous population of *P. vulgaris* in England, first reported by Curtis and Curtis (1985) 38 years ago and recently sampled by Mora Carrera et al. (2021 and present study) is congruent with the assumption of equal viability for S*/S* homostyles. All 11 genotyped homostyles in this population (here named EN6‐M) carry the S*/S*‐genotype (Table 1 and Figure 3); thus, EN6‐M could represent a case in which homostyly increased in frequency over time and became fixed in the population by displacing pins and thrums, as predicted under the assumption of equal viability for S*/S* homostyles (Figure 1d). Alternatively, EN6‐M could have been established by an S*/S*‐homostyle stemming from a nearby population, thus it might have been a monomorphic homostylous population from the beginning. Indeed, Curtis and Curtis (1985) reported that this monomorphic population was located only about 240 m away from a trimorphic population which might have served as a source for the initial homostyle that gave origin to EN6‐M. Finally, EN6‐M had a very low population size (n = 19; Mora‐Carrera et al., 2021), suggesting that stochasticity could have played a role in the fixation of S*/S*‐homostyles in this population and that homozygosity of an *S*‐locus with disrupted *CYP* ^*T*^ might have detrimental effects on population growth.

To summarize, our results suggest that a diploid *S*‐locus with inactivated *CYP* ^T^* may not per se be incompatible with homostyle viability. However, the occurrence of two copies of the remaining *S*‐genes (i.e., *CCM* ^*T*^, *GLO* ^*T*^, *PUM* ^*T*^, and *KFB* ^*T*^) in the genome of a homostyle could have detrimental effects on viability at different stages of the life cycle, possibly stemming from gene‐dosage effects (Ascencio et al., 2021; Li et al., 2015; Rice & McLysaght, 2017; Tasdighian et al., 2017). Future research combining *S*‐locus genotyping and characterization of function and dosage effects of *S*‐genes at different life‐cycle stages with fitness measurements in the field and in greenhouse experiments is essential to address whether differences in viability prevent the widespread fixation of homostyly in *P. vulgaris*.

## AUTHOR CONTRIBUTIONS

**E. Mora‐Carrera:** Conceptualization (equal); data curation (equal); formal analysis (equal); funding acquisition (equal); investigation (equal); methodology (equal); visualization (equal); writing – original draft (equal); writing – review and editing (equal). **R. L. Stubbs:** Data curation (equal); formal analysis (equal); investigation (equal); writing – review and editing (equal). **G. Potente:** Formal analysis (supporting); methodology (supporting); writing – review and editing (equal). **N. Yousefi:** Formal analysis (supporting); methodology (supporting); resources (lead); software (equal); writing – review and editing (equal). **B. Keller:** Investigation (equal); methodology (equal); writing – review and editing (equal). **J. M. de Vos:** Investigation (equal); supervision (equal); writing – review and editing (equal). **P. Szövényi:** Methodology (supporting); software (equal); supervision (equal); writing – review and editing (equal). **E. Conti:** Conceptualization (equal); funding acquisition (lead); project administration (lead); supervision (lead); writing – original draft (equal); writing – review and editing (equal).

## CONFLICT OF INTEREST STATEMENT

The authors declare no competing interests.

## Supporting information


Appendix S1

## Data Availability

Original sequencing reads have been uploaded to the NCBI repository (https://www.ncbi.nlm.nih.gov/) under the BioProject ID: PRJNA1066534. Sequence alignment of all exons of *CYP* ^*T*^ and alignment of *CYP* ^*T*^ promoter region, BAM files of *S*‐locus of the individuals used detect structural rearrangements has been uploaded to Dryad (https://doi.org/10.5061/dryad.prr4xgxtf).
