# Supplementary material for: Genomic analyses elucidate S‐locus evolution in response to intra‐specific losses of distyly in Primula vulgaris
Source: Ecol Evol. 2024 Mar 21;14(3):e10940. doi: 10.1002/ece3.10940 (PMC10955462; doi:10.1002/ece3.10940)
Supplement: Supplementary file 1 — Appendix S1 [file ECE3-14-e10940-s001.docx]

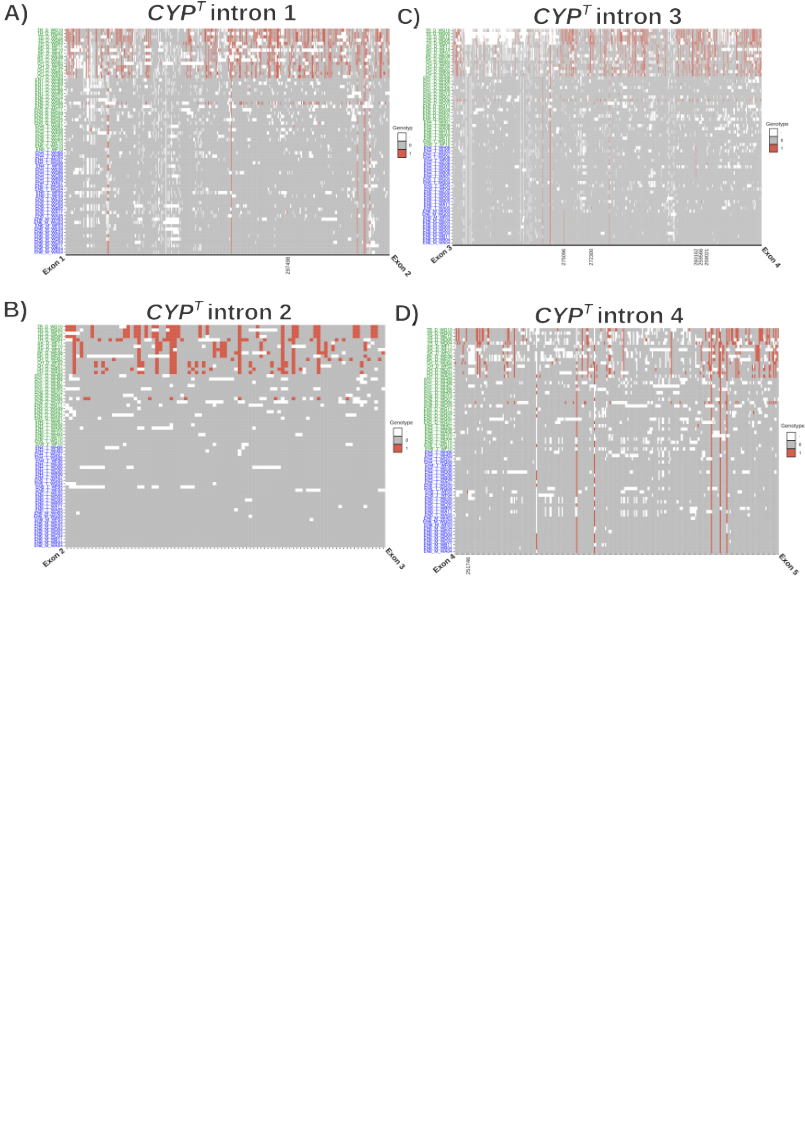


**Figure S1.-** Genetic variability within the four intronic regions of *CYP^T^* from 37 thrums and 31 homostyles (highlighted in green and blue, respectively) of *Primula vulgaris*. The tile plots show sites with variable SNPs in intron A) one (between the first and second exons), B) two (between the second and third exons), C) three (between the third and fourth exons), and D) four (between the fourth and fifth exons) of *CYP^T^*. Invariable sites were omitted. Genotypes are specified in different colors as follows: white indicates missing data; grey indicates that the site is the same as the allele in the reference genome; and red indicates sites that are different from the allele in the reference genome. Only the genomic position of the seven variable intronic SNPs that are exclusive to homostyles (i.e., absent in thrums) within the *S*-locus assembly of *P. vulgaris* (Cocker et al., 2018) is indicated in the x-axis.

**Figure S2.-** *Cis*-re
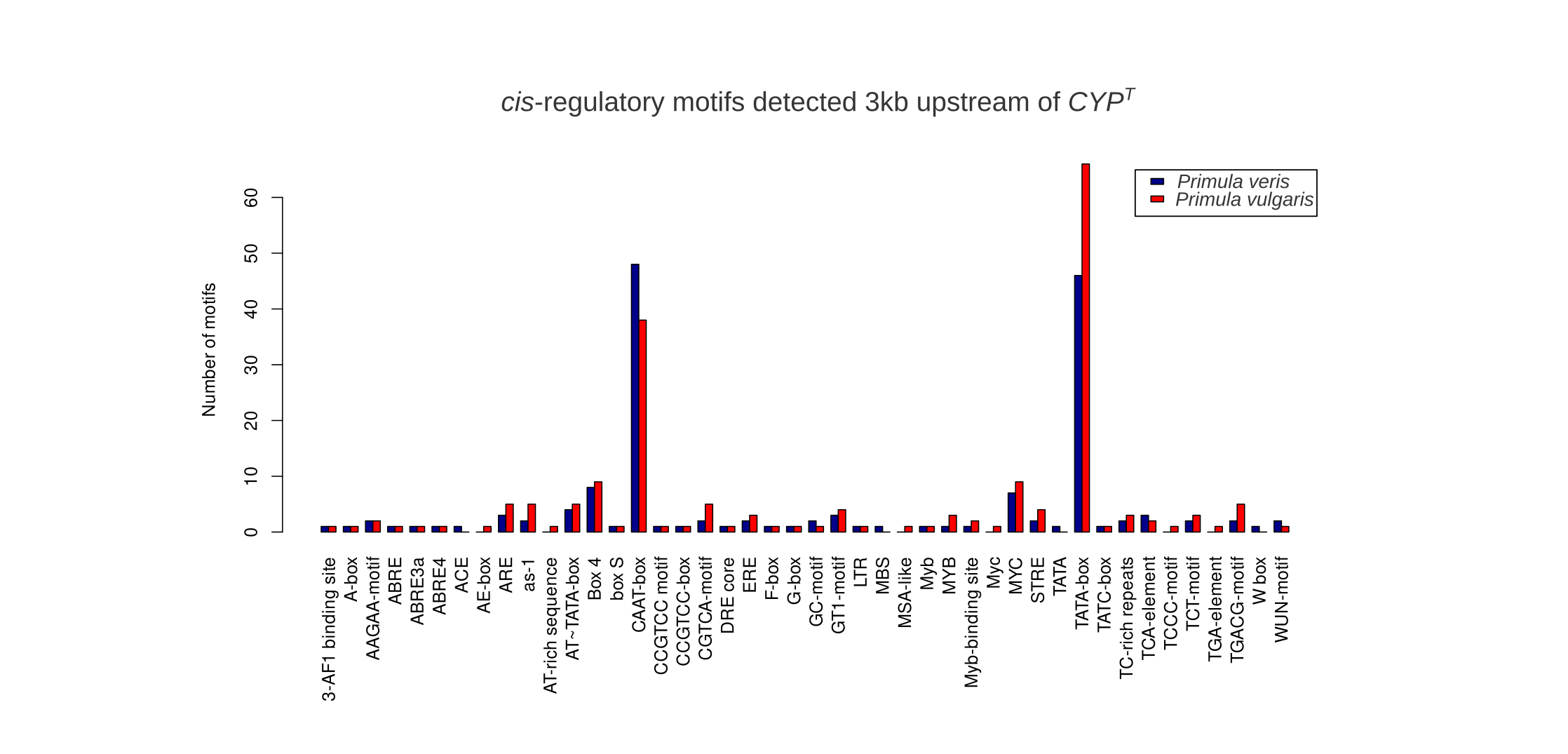
gulatory element motifs identified in the putative promoter region 3kb upstream of *CYP^T^* of *Primula vulgaris* and *Primula veris*. Identification of the regulatory motifs was performed using the PlantCARE database (Lescot et al., 2002).

**Table S1.-** Frequencies of different *S-*locus genotypes (0/0, *S*/0, *S**/0, and *S**/*S**) of *Primula vulgaris* over generations estimated using Crosby’s model (1949) with **A)** lower viability of S*/S*-individuals than *S**/0-individuals and **B)** equal viability between *S**/*S**- and *S**/0-individuals. Each row includes the type (outcrossing [OUT] and sefing [SELF]), combinations, and frequencies of all possible crosses between and within floral morphs in a population. The proportion of viable offspring for each *S-*locus genotype (0/0, *S*/0, *S**/0, and *S**/*S**) in the next generation can be calculated by summing all vertical cells per genotype. To make this formula recursive, it is necessary to readjust the resulting sum of the frequency of each genotype per generation. Viability (specified by ‘v’) refers to the proportion of offspring reaching adulthood, where v = 1 (equal viability) indicate that 100% of the offspring reach adulthood.

**Table S2.-** *S*-locus genomic position of the 24 intronic Single Nucleotide Polymorphisms (SNPs) within *CYP^T^* that segregate in homostyles of *Primula vulgaris*. The number of homostyles and thrums, out of 31 and 37 individuals, respectively, carrying each SNP is indicated. The seven SNPs that segregate exclusively in homostyles (i.e., absent in thrums) is highlighted (see also Figure S1).

**Table S3.-** Estimates of nucleotide diversity at synonymous (π_S_) and non-synonymous sites (π_N_) of **A)** all five *S-*genes (CYP^T^, GLO^T^, CCM^T^, KFB^T^, and PUM^T^) and **B)** their paralogs (CYP734A51, GLO1, CCM1, and KFB1) in three continental populations (TR = Turkey, SK = Slovakia, and CH = Switzerland) and six populations in England (EN). The two trimorphic populations (EN4-T and EN5-T) were further subdivided into distyles (i.e., pins and thrums; DI) and homostyles (HO).
